# WiCHORD+: A Scalable, Sustainable, and P2P Chord-Based Ecosystem for Smart Agriculture Applications

**DOI:** 10.3390/s23239486

**Published:** 2023-11-28

**Authors:** Christos-Panagiotis Balatsouras, Aristeidis Karras, Christos Karras, Ioannis Karydis, Spyros Sioutas

**Affiliations:** 1 Computer Engineering and Informatics Department, University of Patras, 26504 Patras, Greece; akarras@ceid.upatras.gr (A.K.); c.karras@ceid.upatras.gr (C.K.); sioutas@ceid.upatras.gr (S.S.); 2 Department of Informatics, Ionian University, 49100 Kerkira, Greece

**Keywords:** Internet of things (IoT), wireless sensor networks (WSNs), peer-to-peer (P2P) systems, distributed hash tables (DHTs), Chord, LoRa, machine learning, precision agriculture, ubiquitous computing, sustainable water use

## Abstract

In the evolving landscape of Industry 4.0, the convergence of peer-to-peer (P2P) systems, LoRa-enabled wireless sensor networks (WSNs), and distributed hash tables (DHTs) represents a major advancement that enhances sustainability in the modern agriculture framework and its applications. In this study, we propose a P2P Chord-based ecosystem for sustainable and smart agriculture applications, inspired by the inner workings of the Chord protocol. The node-centric approach of WiCHORD+ is a standout feature, streamlining operations in WSNs and leading to more energy-efficient and straightforward system interactions. Instead of traditional key-centric methods, WiCHORD+ is a node-centric protocol that is compatible with the inherent characteristics of WSNs. This unique design integrates seamlessly with distributed hash tables (DHTs), providing an efficient mechanism to locate nodes and ensure robust data retrieval while reducing energy consumption. Additionally, by utilizing the MAC address of each node in data routing, WiCHORD+ offers a more direct and efficient data lookup mechanism, essential for the timely and energy-efficient operation of WSNs. While the increasing dependence of smart agriculture on cloud computing environments for data storage and machine learning techniques for real-time prediction and analytics continues, frameworks like the proposed WiCHORD+ appear promising for future IoT applications due to their compatibility with modern devices and peripherals. Ultimately, the proposed approach aims to effectively incorporate LoRa, WSNs, DHTs, cloud computing, and machine learning, by providing practical solutions to the ongoing challenges in the current smart agriculture landscape and IoT applications.

## 1. Introduction

Interconnected devices are now becoming even more popular to simplify and enhance various applications in the context of Industry 4.0. Some major examples of smart interconnected devices utilization are smart cities, smart industry, and smart agriculture applications. In these applications, a set of devices collecting and processing data communicate with each other to fulfill the purposes of each application. For instance, in a smart city ecosystem, the smart infrastructure equipment interacts with the corresponding sensors on smart vehicles to improve the autonomous vehicle driving experience and safety. All the above applications are enabled by technologies such as the Internet of things (IoT), cloud computing, and artificial intelligence (AI). The solution proposed in this work combines the preceding technologies with particular emphasis on smart agriculture applications.

Smart agriculture is the process of incorporating smart technologies and interconnected devices into agriculture, in order to improve the production yield both in terms of quality and in terms of quantity, by utilizing data collected from sensors and smart devices deployed on the field. Some common examples of smart agriculture practices are smart irrigation systems, crop monitoring systems, and plant disease prevention mechanisms, among others [1]. An example of a smart agriculture application is the utilization of sensors along with the technologies of the Internet of things and machine learning for agricultural produce growth monitoring and plant disease forecasting purposes [2]. In essence, the benefits of applying smart agriculture techniques are multiple, with farmers saving valuable time and resources while consumers have real-time access to quality agricultural products.

One fundamental technology supporting smart agriculture is the Internet of things. In the concept of the Internet of things, every device possible, beyond personal computers, has computational capabilities along with internet connectivity [3]. These IoT devices might be sensors and smart appliances in a smart home or in a smart city environment. The concept of the IoT can be applied in a plethora of use cases such as smart industry, smart cities, and smart agriculture [3]. This results in real-time monitoring and minimized human intervention on various tasks via data acquisition, process automation, and device control over long distances.

The Internet of multimedia things (IoMT) [4] represents an evolutionary step beyond traditional IoT by integrating various sensors and devices capable of collecting and processing multimodal information, such as audio, video, and sensor data. The IoMT expands the horizons of data collection and process automation by enabling devices to not only capture traditional data like temperature and humidity but also engage with rich multimedia content. This advancement allows for a deeper understanding of the environment, enabling applications in areas like smart cities, healthcare, industrial automation, and agriculture. The ability of the IoMT to harness and process diverse data types offers a powerful foundation for informed decision-making and innovative solutions in an increasingly connected and multimedia-rich world.

An important chapter in the IoT research is the utilization of wireless sensor networks (WSNs). A wireless sensor network is a key component for many IoT applications and consists of a group of sensor devices measuring local environmental conditions and acting as nodes. These sensor devices are deployed over a large field either randomly or following a predefined topology and they forward their sensor readings to the gateway node of the network for further data processing. Each sensor node device is equipped with a microcontroller unit (MCU) along with a wireless communication module for data transmission in the network, and various sensors are used for local conditions’ monitoring [5]. Wireless sensor networks are utilized in various applications such as smart energy grids, environmental monitoring, and smart cities [6].

For the support of the communication between the nodes on a wireless sensor network, different wireless technologies are used depending on the application. On the one hand, short-range wireless technologies such as Wi-Fi and ZigBee support the deployment of a wireless sensor network on a smaller scale with a maximum wireless range of 100 m [7]. For example, a short-range wireless sensor network can be deployed on a smart industry facility. On the other hand, long-range wireless technologies such as LoRa, NB-IoT, or other LPWAN technologies support wireless communication over long distances with low energy consumption. On a long-range wireless sensor network, the distance between two nodes can reach up to a few kilometers [7]. These long-range wireless communications are utilized in applications such as precision agriculture, in which battery-powered devices are deployed over large agricultural fields, and therefore, as we present a smart agriculture use-case, this work focuses on large-scale LoRa wireless sensor networks covering long distances.

On a wireless sensor network, data are forwarded in packets or messages between the nodes of the network in order to reach the gateway node. For efficient data forwarding achievement, a routing protocol must be implemented on the wireless sensor network [8]. For data routing purposes in the wireless sensor network model proposed in this work, the Chord protocol for peer-to-peer (P2P) systems [9,10,11] was selected. Chord is a distributed hash table (DHT), in which any hashed key belongs to a fixed circular hash space with *m*-bit addresses and $2^{m}$ maximum keys [9,10]. This hash space contains nodes and keys, with the basic Chord property dictating that each key is mapped on its successor node on the ring [9]. Each node on the network is only aware of its successor and predecessor node along with a set of a few other nodes on the network, also known as “fingers” [9]. Chord is a self-stabilizing model that supports node joins and departures on the network while maintaining stability and operation [9], making it ideal on a wireless sensor network use case in which nodes are powered by batteries and may leave the network if a battery runs out. A very important operation of Chord is the support of lookup queries. On the execution of a lookup query, Chord retrieves efficiently the corresponding node that holds the requested key by forwarding the query to the other nodes of the network. The Lookup query on the Chord protocol can be resolved in logarithmic computational complexity.

In this work, the initial methodology of the WiCHORD protocol from [12], which is an adaptation of the Chord protocol from [9] for P2P LoRa wireless sensor networks, is extended in detail in order to implement an overlay for wireless sensor networks. These sensor networks are suitable to be deployed in precision agriculture applications, where the benefits of WiCHORD such as its energy efficiency, network stability, and sensor node lookup query efficiency can be utilized due to the nature of such applications. Additionally, a representative case study of the utilization of the proposed extended WiCHORD protocol for wireless sensor networks in a precision agriculture ecosystem is presented. This case study is an applications ecosystem by the name “VineLink Monitoring” that can utilize the proposed WiCHORD overlay in agriculture. The application ecosystem includes the implementation and deployment of a LoRa wireless sensor network, suitable for the WiCHORD protocol overlay, over a vineyard field located in Central Greece, which monitors the environmental conditions of the vineyard using the onboard sensors in real time. Then, the recorded data are stored in a database on the cloud to be presented to the user via a dedicated Web dashboard application. Finally, the recorded sensor data consisting of value pairs of vineyard temperature and humidity are used to train some machine learning models capable of predicting the occurrence and incubation period of the grape downy mildew plant disease that is quite common in vineyards.

The contributions of this work are the following. First, this work extends and implements a P2P distributed overlay for wireless sensor networks from [12], based on the Chord protocol from [9], which ensures the stability of the wireless sensor network when sensor node devices join or leave the network. Also, the proposed protocol from [12] extended in this work can perform efficient lookup queries to locate a given sensor node device within the sensor network. Due to the decentralized nature of the proposed solution, it is considered that the execution of the join, leave, and lookup operations of the WiCHORD protocol from [12] on a wireless sensor network only uses a small subset of the total network nodes, and therefore, it is deemed that the energy consumption of the devices on the wireless sensor network is significantly reduced, which is important since the sensor devices of the network rely mostly on batteries for power. Apart from the reduction in the energy consumption on the wireless sensor network, the lookup operation of the WiCHORD protocol from [12] lets the user efficiently query a given sensor node device by its unique MAC address and retrieve the current sensor data and status of this sensor node device. In essence, the aim of this work is to extend the methodology of [12] and verify its efficiency via simulation.

The structure of the remainder of this work is as follows. Initially, in Section 2, a literature review on the fields of the Internet of things, big data systems, and smart agriculture systems is performed. Next, in Section 3, the proposed methodology of an enhanced adaptation of the Chord P2P protocol on wireless sensor networks using LoRa technology is presented. In Section 4, a representative case study of utilizing the proposed Chord-based wireless sensor network on a precision agriculture application in vineyards is shown. Section 5 presents simulation results regarding the performance of the proposed Chord-based WSN model. Section 6 presents the discussion regarding this work. Finally, in Section 7, concluding remarks and future directions of this work are given.

## 2. Background and Related Work

This section presents a comprehensive literature review of the sources explored during the research conducted within the context of this study. The main focus of this study is to investigate the innovative applications and infrastructures in agriculture, specifically emphasizing the integration of the Internet of things, peer-to-peer (P2P) systems, and machine learning (ML) techniques. To provide a comprehensive analysis, the bibliographic references are organized into subcategories that connect with the key knowledge domains related to the conjunction of agriculture, IoT, P2P systems, and ML progression.

### 2.1. Peer-to-Peer Systems and Internet of Things

In exploring the domain of peer-to-peer (P2P) systems, a detailed literature review was undertaken, beginning with themes of a general nature that pertained to P2P systems such as distributed hash tables (DHTs) and extending to the incorporation of P2P systems in the context of IoT.

To elaborate, as previously described, a P2P network is characterized by a network topology where all participating nodes, or peers, share equivalent responsibilities and collaborate by sharing computational resources [13]. Several widely used applications of P2P systems include the implementation of routing protocols to facilitate content discovery and sharing across nodes, as well as the application of load-balancing strategies to distribute workload equitably among participating nodes [14,15]. In the context of P2P applications involving content sharing, the concept of routing becomes crucial. Routing pertains to the process whereby a search query must be reliably addressed with respect to the node that hosts the requested data [14]. Following this, the principle of load balancing involves the fair assignment of stored objects across the nodes of a P2P system, as mentioned in [15].

A particularly noteworthy subclass of P2P systems encompasses distributed hash tables (DHTs) [16]. Unlike conventional hash tables, DHTs function by storing keys across nodes within a dynamic network. The dynamic nature of distributed hash tables (DHTs) permits nodes to seamlessly join or leave the network anytime, ensuring stability, while also facilitating key lookup queries distributed among the nodes [15]. Examples of widely implemented DHT structures include but are not limited to Chord, Pastry, Kademlia, CAN, and Tapestry [16]. This work focuses primarily on the distributed hash table known as Chord [9], where a modified and advanced version has been developed for wireless sensor networks, as discussed in [12]. However, in addition to Chord, various significant DHT algorithms were also considered. For example, Pastry [17] operates similarly to Chord in its mapping of keys to nearby nodes and in its requirement for each node to only be aware of a subset of the other nodes in the network. Contrarily, Kademlia [18] differentiates itself from Chord in its method of calculating the distance between two nodes in the network by using the binary XOR operation [16].

The adaptation of distributed hash tables from peer-to-peer systems to applications within the Internet of things, such as wireless sensor networks (WSNs), presents a topic of considerable interest, particularly in serving purposes such as routing messages among sensor nodes [19]. At this point, various efforts have been made, with a particular focus on the Chord protocol for distributed hash tables being applied in wireless sensor networks. The inaugural application of a sensor network utilizing the Chord protocol, as introduced in [20], involves a scheme where sensor nodes are grouped into clusters based on physical location, with the cluster head selected by the other nodes in the cluster based on energy levels. In the application of Chord within this sensor network, as described in [20], the cluster nodes act as keys, with the cluster heads operating within Chord’s circular hashing space. This results in a streamlined routing of messages from the individual sensor nodes, via the cluster heads, directly to the base station, serving as the final destination.

A similar approach by the same authors as [20], considering the energy levels of sensor nodes, is presented in [21]. This approach reiterates the relevance of energy efficiency, further highlighting the potential for the utilization of distributed hash tables, and the Chord protocol specifically, within wireless sensor networks.

In [22], an innovative application of the Chord protocol is introduced, designed to facilitate the connection between a sensor network and the Internet for executing search queries. This method effectively leverages Chord’s inherent properties while simultaneously adopting a two-tiered hierarchy within the sensor network, organizing nodes into primary and secondary tiers, to optimize the routing of search queries across the network.

The study conducted by the researchers in [23] takes inspiration from the Chord protocol to design an ingenious peer-to-peer routing scheme. This novel mechanism introduces significant enhancements in terms of data management operations. It not only supports traditional functions such as updating and deleting data within the sensor network but also ushers in a fresh perspective by suggesting an organizational structure for the sensor network based on the nodes’ physical positions. This location-based organization of nodes potentially facilitates more efficient communication, data sharing, and network management, promising a significant step forward in the domain of sensor networks.

Expanding on the varied utilization of the Chord protocol, ref. [24] presents a novel application in the realm of sensor networks, spatial positioning of objects, and edge computing. The conceptualized system utilizes sensor networks to precisely determine the geographical location of various objects, while edge computing provides the computational energy required for rapid, localized data processing. Following a similar trajectory, ref. [25] introduces an effective utilization of peer-to-peer systems for wireless sensor networks. This approach encompasses the facilitation of data exchange—both transmission and reception—amongst the network nodes. The introduction of this system represents an essential progress in wireless sensor network communication, as it promotes efficiency and robustness while ensuring seamless data exchange. These innovations, as proposed by [24,25], signify the potential and versatility of the Chord protocol in various applications, contributing meaningfully to the evolving landscape of IoT technologies.

The application of distributed hash tables in wireless sensor networks has been a popular solution for data routing, especially in the context of IoT environments. However, these systems represent only a facet of the wide array of possible applications of peer-to-peer (P2P) technologies within wireless sensor networks. The energy efficiency concern within these networks, for example, is addressed by a methodology proposed in [26]. This approach enables a node within the sensor network to locate and delegate a query to other nodes possessing higher energy levels, especially when the querying node’s energy state is nearing depletion. Thus, this method aids in preserving the sustainability of the network’s energy resources, which is paramount for the longevity and efficiency of sensor networks. Importantly, the foundation of this energy management method owes its conception to the methodologies detailed in [27], demonstrating the interconnected evolution of P2P solutions within sensor networks.

Ultimately, peer-to-peer systems have a significant potential and can be utilized in a wide range of ways, especially in the context of Internet-of-things applications. As a case in point, ref. [28] proposes the development of an IoT application that exploits peer-to-peer systems. This not only demonstrates the utility of such systems but also opens a promising avenue for future exploration in this dynamic area.

### 2.2. Machine Learning Techniques in Precision Agriculture and Agricultural Sector

The integration of machine learning (ML) techniques into the agricultural sector heralds a transformative era marked by precision and resilience. The combination provides an effective strategy for addressing some of agriculture’s most complex problems, such as the hazards of climate change, the increasing population, and the continuing difficulty of guaranteeing global food security. One key area where ML finds substantial application is precision agriculture (PA), a sophisticated approach that employs ML algorithms to scrutinize extensive and varied datasets derived from disparate sources such as satellite images, soil sensors, weather forecast systems, and drone technology. This data analysis underpins evidence-based decision-making, empowering agriculturists to streamline resource distribution, magnify crop yields, and minimize detrimental environmental impacts.

For example, ML can provide accurate crop yield predictions and early warnings for potential pest invasions or disease outbreaks, facilitating preventive measures and reducing potential losses. Moreover, within the realm of resilient agriculture, ML plays a crucial role in forecasting the impact of climate fluctuations on agricultural output. These predictive insights serve as the basis for the creation of adaptive farming practices and the cultivation of resilient crop species, fostering an agricultural system robust enough to withstand the unpredictable nature of climatic shifts. Therefore, the interplay between ML techniques and agriculture presents a profound opportunity to significantly improve the effectiveness and resilience of global agricultural systems, becoming an indispensable component of sustainable agricultural development.

One of its noteworthy applications is precision agriculture (PA), where ML algorithms diligently analyze a diverse range of data derived from a multitude of sources such as satellite imagery, soil sensors, weather forecasting systems, and drones. By processing this vast corpus of data, ML enables informed decision-making in agriculture, promoting the effective use of resources, amplifying crop yields, and reducing environmental footprint. Furthermore, it facilitates the prediction of crop yields with a remarkable accuracy and provides early warnings for potential pest invasions or disease outbreaks, which allows for preemptive actions and the mitigation of potential losses [29].

Similarly, unmanned aerial vehicles (UAVs) have become an indispensable tool for precision agriculture. Their ability to gather real-time data has proved beneficial for various agricultural tasks such as spraying, monitoring, yield estimation, and weed detection [30]. While their usage has been extensive in outdoor farming, opportunities for their application in controlled environments such as greenhouses remain largely untapped. The advent of advanced wireless sensor network (WSN) technologies, such as LoRaWAN, has opened up new horizons for precision agriculture in greenhouses by providing sustainable solutions for resource management and yield quality enhancement [31].

For instance, Goel et al. highlight the significance of precision agriculture, demonstrating how machine learning algorithms can automate farmers’ expertise to enhance field management and increase crop yield [32]. This method involves data collection from various instruments such as sensors, drones, and other advanced devices, followed by the integration of diverse ML algorithms for informed decision-making. With these innovations, farmers can monitor their fields in real time, quickly detect weeds and insects, schedule watering more efficiently, and much more. By optimizing resources and increasing productivity, precision agriculture is instrumental in helping farmers meet the growing global demand for food. In this domain, machine learning offers solutions for crop and soil management, weather prediction, efficient water use, and even postharvest procedures. Also, another example of machine learning and data mining techniques utilization in a greenhouse is shown in [33].

Edge intelligence and TinyML, underscored by recent studies [34,35,36], emerge as essential technological improvements with the potential to significantly reshape precision agriculture, increasing its efficiency and productivity. By processing data locally, edge computing systems not only enhance the reliability of complex communication protocols but also encourage further improvements in precision agriculture methodologies. This integrative approach to data processing and analysis ultimately uplifts the precision agriculture paradigm [34].

The combination of AIoT with modern technologies, notably smart sensors, and cloud computation, represents a paradigm shift towards improved agricultural techniques [37]. AgriFusion, as detailed in [38], is an illustrative architectural framework that incorporates the combination of IoT and emergent technologies based on a comprehensive survey of precision agriculture. Furthermore, unmanned aerial vehicles (UAVs) are essential to collecting detailed field imagery, with edge intelligence providing a prompt data analysis [39]. In addition, TinyML’s ability to incorporate AI within sensors provides solutions for energy conservation while addressing common privacy, security, and latency issues, as well as reducing the sensor’s battery consumption [40]. These kinds of implementations also serve security purposes, as demonstrated by their capacity to identify animals near farmland [41]. In essence, using edge intelligence together with TinyML can lead to better food production, make farming practices more efficient, and support a more sustainable agricultural industry.

While the integration of AIoT and TinyML offers foundational improvements to agricultural processes, there are also specialized advancements in machine learning that further refine precision agriculture techniques. Adding another layer of sophistication, novel techniques like “LettuceTrack”, a state-of-the-art multiple-object tracking (MOT) method, have been developed. This method has the ability to simultaneously detect and track individual plants, aiding the precise application of liquid fertilizer and pesticide through agricultural robots. It effectively reduces the quantity of chemicals sprayed, presenting a more economical and environmentally friendly solution compared to conventional indiscriminate spraying methods [42]. Such innovative ML applications underscore its potential in shaping a more resilient and efficient agricultural sector.

### 2.3. Applications of Machine Learning Algorithms in Agriculture

The use of machine learning techniques and algorithms in various sectors has seen a remarkable surge, largely due to their potential to extract insightful knowledge from data. The unique demands of the problem at hand and the anticipated outcome drive the selection of the technique, such as regression, clustering, and classification, among others [43,44]. This recognition of machine learning’s diverse array of techniques and algorithms is a pivotal stepping stone in comprehending its profound role and transformative potential in the field of agricultural technology.

Various machine learning algorithms have gained widespread application in the agricultural sector, with each algorithm demonstrating unique functionalities while providing notable contributions. This extensive collection encompasses decision tree models, such as the random forest, extending to complex neural networks that emulate human cognitive functionalities. These pioneering algorithms have played a pivotal role in effecting a transformative overhaul of agricultural practices, thereby facilitating marked enhancements in productivity and operational outcomes.

The adaptability of machine learning techniques is demonstrated by a particular adaptation of the well-known k-nearest neighbors (k-NN) algorithm. Traditionally, k-NN is employed for categorizing data within a dataset based on the relative distances among different samples within the set. In a distinctive approach [45], the conventional k-NN model is aptly customized to accommodate multidimensional data, thus broadening its applicability to cater to more complex data structures. This ability to adjust and adapt to varied data structures underscores the versatility of machine learning as a potent tool for data analysis and interpretation.

The random forest algorithm, a preeminent decision tree model, has been harnessed extensively within the agricultural sector, particularly in the areas of crop yield prediction and plant disease detection. This ensemble learning methodology constructs a multitude of decision trees during the training phase, culminating in a prediction that reflects either the mode of the classes (classification) or the mean prediction (regression) of the individual trees [46]. A distinct asset of the random forest algorithm is its proficiency in decoding intricate patterns hidden within complex, multivariate agricultural datasets, thereby enabling accurate predictions of pivotal outcomes such as crop yield or disease manifestation [46]. This aptitude equips random forest with the capacity to provide an unparalleled degree of precision and predictive power in the domains of agricultural planning and disease control.

Further empirical evidence corroborates the robust applicability and remarkable versatility of the random forest algorithm within the agricultural landscape. Distinct studies bear testament to this assertion. In a similar scope, Wu’s study showcased the dexterity of this algorithm by successfully extracting and mapping diverse forest types in Northern China utilizing an array of multisource data [47]. Notably, a study by Kumar et al. utilized random forest for the ambitious task of predicting soil fertility. The results not only graded soil for crop recommendation but also signaled a paradigm shift towards data-driven, predictive agriculture [48].

### 2.4. Smart Agriculture Applications for Vineyards and Wineries: Types, Operations, and Benefits

The integration of smart agriculture applications is increasingly becoming essential for winery operations, offering a range of operational benefits. Such tools provide valuable insights into real-time environmental factors like vine health and soil moisture. As a result, wineries can make more accurate decisions about irrigation, fertilization, and harvest timings [49]. Furthermore, precise resource management, encompassing the optimized use of water, fertilizers, and pesticides is possible, leading to a reduction in waste and an increase in yield [50].

Recent studies highlight the transformative role of smart agriculture in the winery sector. Tardáguila’s research et al. delve into the use of sensing technologies combined with artificial intelligence, illustrating their potential in optimizing grape cultivation and refining wine production processes [51]. Sarri et al. introduce precision agriculture methods, suggesting they can be crucial in enhancing wine farm management [52]. Building on this, Oreški et al. highlight the avenue of employing machine learning techniques, especially in predicting wine quality [53]. Furthermore, Vela et al.’s study underscore the significance of conducting energy audits in wineries [54]. They propose that there are opportunities for energy conservation, notably through the adoption of efficient chillers and the integration of adjustable-speed pumps [54].

Applying the capabilities of the use of precision agriculture vineyard and winery-specific intelligent agriculture techniques improves production quality and efficiency in operation. This transformation depends extensively on the Internet of things, automation, image analysis, artificial intelligence, and robotics [55,56,57]. Expanding our knowledge of the various applications, we can cite the following:

- **Vineyard health insights**: IoT sensors strategically deployed provide in-depth insights into the health and vitality of vineyard produce. This detailed information aids producers in optimizing various phases of production, resulting in more effective and targeted vineyard treatments [55].
- **Environmental parameter analytics**: Cost-effective IoT devices collect crucial environmental data points, such as temperature, humidity, and soil moisture. This information is subsequently incorporated into complex algorithmic models designed to predict and prevent potential agricultural diseases [58].
- **Automated vineyard navigation**: Autonomous navigation of robots in vineyards poses unique challenges due to the need to avoid damaging grapes. A newly proposed algorithm addresses this challenge by leveraging Lidar sensor data and not relying on GPS. The algorithm enables safe robot navigation in vineyards by deftly handling dynamic obstacles, such as moving people, and ensuring that the robot’s positions remain smooth without sudden stops or sharp turns. Tests in both simulations and actual vineyards have confirmed its efficiency, suggesting its potential for smooth integration into vineyard robots [59]. Machine learning and computer vision can also be combined to enable autonomous navigation of vineyards using only camera sensors. This method is valuable for vineyards, orchards, and other agricultural settings [60]. In addition, a vision-based autonomous navigation system was developed for agricultural robots to navigate in trellised cropping systems, such as vineyards. The framework consists of three main components: environment perception, robot localization, and path planning [61].
- **Real-time water status monitoring**: Utilizing a LoRaWAN IoT framework, vineyards can now obtain real-time information and measurements on plant hydration levels. This technology promises more precise crop management and practical use of water resources, leading to more sustainable agriculture [62]. All in all, smart agriculture systems and their methodology is deemed as a tool that will, among other effects, increase the effectiveness of the local administrations for protection, conservation, and sound use of water resources and at the same time will improve operational reliability and safety of water supply systems, especially in touristic areas, thus leading to a more sustainable use of water resources [63]. In addition, Alves de Melo et al. present an innovative and automated solar-powered soil moisture sensor designed to optimize water management in agriculture [64]. The study offers a detailed overview of the sensor’s design, construction, and its impressive data-gathering capacities. The authors highlight the numerous advantages of this cutting-edge technology, notably its potential to enhance irrigation efficiency and minimize water wastage. Yet, they also openly address the potential challenges and constraints of integrating this solution across varied agricultural landscapes. The research essentially acts as a key element in soil moisture monitoring technology, highlighting its critical importance for enhancing sustainable water management in agriculture. Also, Bertocco et al. introduce an advanced methodology for measuring volumetric water content (VWC) in soil, utilizing RSSI-based augmented sensors combined with machine learning algorithms [65]. Analyzing a comprehensive dataset of 27,000 data points, which comprises readings from affordable sensors, RSSI measurements via the LoRaWAN gateway, and standard VWC values, the authors highlight the method’s adaptability and effectiveness across different soil compositions. By emphasizing the technique’s potential for improved accuracy, the study showcases its importance as a significant step forward in agriculture and environmental monitoring within the IoUT landscape.
- **Efficient aerial spraying routes**: Precision agriculture is an emerging field that aims to increase the efficiency and productivity of agriculture while minimizing the use of resources such as water and pesticides. One area of focus in precision agriculture is the development of autonomous pesticide-spraying robots, which can reduce the exposure of farmers and farm workers to harmful pesticide chemicals [66,67,68]. In addition to the development of autonomous pesticide spraying robots, another way to improve the efficiency and productivity of agriculture is to use the traveling salesman problem (TSP) and Theta algorithm to create unique trajectories for unmanned aerial systems (UAS). These paths ensure a precise aerial distribution of plant protection products (PPP) in vineyards, leading to better quality control, financial efficiency, and reduced environmental impact [69].

The widespread utilization of these smart agricultural tools provides vineyards and wineries with a plethora of benefits. Additionally, there is a notable increase in yield, productivity, and sustainability. Moreover, the integration of technology alongside traditional agricultural techniques reduces costs and reduces dependency on manual labor. Furthermore, this incorporation promises a more environmentally friendly approach, minimizing resource waste and encouraging ecofriendly practices. This sustainable approach not only increases the economic viability of vineyards but also ensures their longevity, ensuring future generations of consistent, high-quality wine production.

#### Plant Diseases in Vineyards

Vineyards are complex ecosystems that are home to a diverse range of species. This delicate balance can be disrupted by a variety of diseases that affect the grapevine, most of which are caused by pathogenic fungi. These diseases significantly diminish the quality and quantity of the grape yield, which can have a negative impact on wine production and the overall health of the vineyard.

Vineyards face the challenge of numerous plant diseases that can significantly harm the health of grapevines and reduce harvest yields. Among these, downy mildew and powdery mildew are notably prevalent. Downy mildew results in leaf wilting and shedding, compromising the plant’s photosynthetic capacity and, consequently, sugar production. On the other hand, powdery mildew manifests as a white film on the leaves, obstructing sunlight and impeding photosynthesis. Beyond their direct effects, these diseases can render grapevines more vulnerable to other infestations and disorders. The effects of plant diseases go beyond the visible damage. They can cascade to impact the quality of the produce, even if the produce appears healthy. For example, downy mildew can cause leaf loss, which reduces sunlight exposure and can lead to reduced sugar content in grapes, affecting the wine’s overall quality.

Vineyard sustainability and productivity over the long term depend on a detailed knowledge of plant diseases and how to manage them. Researchers are constantly developing new and more effective methods for preventing and controlling plant diseases in vineyards, such as using resistant varieties, cultural practices, and biological control agents. The adoption of such informed strategies is crucial to ensuring the long-term sustainability and productivity of vineyards worldwide. Vineyard owners can potentially protect their crops and ensure a healthy and productive harvest for years to come by taking steps to prevent and control plant diseases.

Vineyards around the world are constantly under threat from a range of plant diseases, which have been the research topic of a plethora of academic studies. Table 1 provides an overview of common diseases affecting vineyards, which are caused by factors such as temperature, humidity, rainfall, etc., that can be monitored using corresponding data acquired from sensors deployed on the field. This study can serve as a crucial resource for vineyard farmers and researchers aiming to deepen their understanding and devise effective countermeasures against these harmful diseases. Notably, our study primarily focuses on the prevention of grape downy mildew (GDM). This disease in particular is detected and identified using our advanced sensor networks. Specifically, since grape downy mildew is influenced by the local temperature and humidity of the affected vine, these environmental conditions can be monitored using our implemented sensor networks and the disease can be predicted utilizing machine learning techniques. The prediction of grape downy mildew using our implemented sensor networks for this study is presented in detail in Section 4 of this work.

### 2.5. State-of-the-Art Comparison

In the context of the Internet of things and smart agriculture, a series of studies have systematically investigated the fusion of the Internet of things (IoT) with wireless sensor networks (WSN), revealing advancements in how agricultural resources are managed and decisions are made. The collective body of work transitions from basic communication protocols to complex systems specifically designed for agricultural needs. Notably, the Chord protocol features prominently, with its deployment in peer-to-peer (P2P) networks improving the robustness and effectiveness of data sharing in the context of agriculture.

Table 2 offers a clear comparison of different studies, showing the key areas of focus, the technologies used, and the use of Chord-based protocols in smart agriculture systems. This overview highlights the growing use of IoT and WSN technologies, especially the role of Chord-based designs in making smart agriculture more efficient and scalable.

## 3. Proposed Methodology

In this section, the proposed extensions to the methodology of the WiCHORD Protocol from [12], which acts as a Wireless sensor network overlay, is presented along with a use-case scenario to apply this protocol on P2P LoRa wireless sensor networks utilized in smart agriculture applications. First, a brief introduction is given to the WiCHORD Protocol as originally proposed in [12] along with the ideal model of wireless sensor networks for this overlay solution in precision agriculture. Then, the context of machine-to-machine (M2M) communication between the sensor node devices on a sensor network running the WiCHORD Protocol is defined. Next, the benefits of optimizing the WiCHORD Protocol from [12] for WSNs is discussed. Finally, a representative use-case scenario for efficient node lookup on a sensor network is proposed.

Before diving into the concept of the WiCHORD protocol, let us first explain the fundamental elements of WSNs and in particular sensor nodes and gateway nodes.

**Definition** **1.** *Sensor node: A sensor node, often simply referred to as a node, is a small electronic device within a WSN that performs the primary function of collecting data from the environment. Each sensor node is typically equipped with one or more sensors, a microcontroller processing unit (MCU), memory, a power source, and a wireless communication component. These nodes are responsible for capturing specific parameters such as temperature, humidity, soil moisture, or other environmental indicators. They process this information and then transmit it to a base station or gateway node for further processing, analysis, or remote access.*

**Definition** **2.** *Gateway node: A base station or gateway node acts as a bridge between the sensor nodes and a larger network, such as a local area network (LAN) or the Internet. It gathers the data transmitted by the sensor nodes and then relays this information to a central system for storage, analysis, or real-time monitoring. Gateway nodes often have more robust processing capabilities, higher power sources, and broader communication technologies than individual sensor nodes. They serve as the communication endpoint for sensor nodes that may not have the ability to directly connect to the broader network.*

In the operation of WSNs within smart agriculture, sensor nodes are dispersed throughout the agricultural field to monitor various conditions affecting crop growth. The gateway node collects the data from all sensor nodes and can either store them for local decision-making or send it to a remote server or cloud service, where advanced analytics can be performed, often involving machine learning algorithms for predictive analysis and decision support.

### 3.1. Introduction to the WiCHORD Protocol

The WiCHORD protocol as initially proposed in [12], is a modified version of the Chord protocol from [9], adapted for efficient data routing and stability on a P2P wireless sensor network. The key motivations of applying this Chord-based protocol on a wireless sensor network to act as a network overlay are the following: First of all, Chord ensures the overall stability of a network when various nodes join or leave the network. This is useful on a sensor network paradigm in which node devices run on batteries and may disconnect from the network if their battery gets low [12]. Additionally, Chord supports lookup queries with a logarithmic computational complexity, and this can be exploited for a requested sensor node to be located efficiently within the sensor network [12], but this also results in achieving an overall energy efficiency on the network since only very few subsets of nodes are involved in resolving the lookup query. On top of that, since each sensor node is aware of only a few other nodes on the sensor network running the proposed Chord overlay, only a few node IDs are stored in the node’s memory resulting in a higher storage efficiency which is crucial for sensor node devices with limited memory capacity.

In the next subsections, the ideal architecture for a wireless sensor network model suitable for the WiCHORD protocol to run on top of is described. Also, the WiCHORD protocol, as initially proposed in [12], is introduced in an algorithmic manner.

#### 3.1.1. Ideal Sensor Network Model

The wireless sensor network model which is ideal for WiCHORD to run as an overlay on top of has the following structure. First of all, the wireless sensor network is comprised of simple sensor nodes and gateway or base station sensor nodes [12]. Each simple sensor node device consists of a microcontroller unit (MCU) chip such as the ESP32 from Espressif Systems along with various sensors to monitor the local environment, a LoRa transceiver module for data packet exchange among the other nodes on the network over long-range distances, and a battery for power. After collecting data from the onboard sensors, the simple sensor node device forwards these data in packets towards the gateway sensor node of the network, as stated in [12] and in the corresponding above-mentioned definition. The gateway sensor node of the network has a similar architecture and functionality to those of the simple sensor node, with additional internet access in order to relay all the received sensor data to the user over the internet. Also, the gateway node enables the user to manage the sensor network and run queries via the WiCHORD protocol on the sensor network, as stated in [12] and in the corresponding above-mentioned definition.

By design, each time only one of the available gateway nodes acts as the gateway node of the WiCHORD sensor network, and in case this node device fails, another gateway node from those available can be elected to replace it. All the remaining gateway node devices that are not elected to run as the base station of the sensor network are running as simple sensor node devices [12]. For a wireless sensor network running WiCHORD, the total number of running simple sensor node devices must be located within the LoRa wireless communication range, in order to be able to exchange data packets between themselves [12].

**Figure 1:**
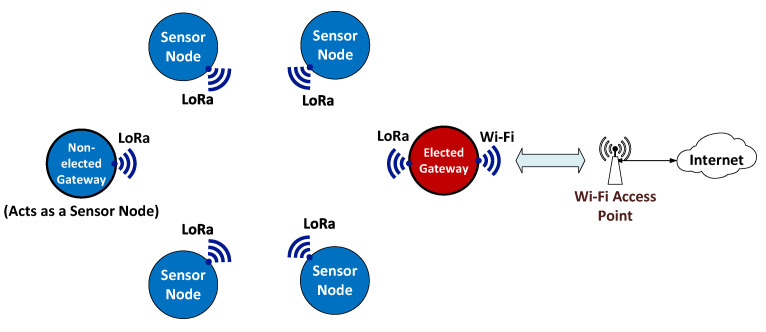
The architecture of the ideal WSN model for the WiCHORD protocol. (Inspired from [12]).

The proposed ideal wireless sensor network model for the WiCHORD protocol to act as an overlay on top of is shown in Figure 1, as described above.

#### 3.1.2. Brief Description of the WiCHORD Protocol

**Figure 2:**
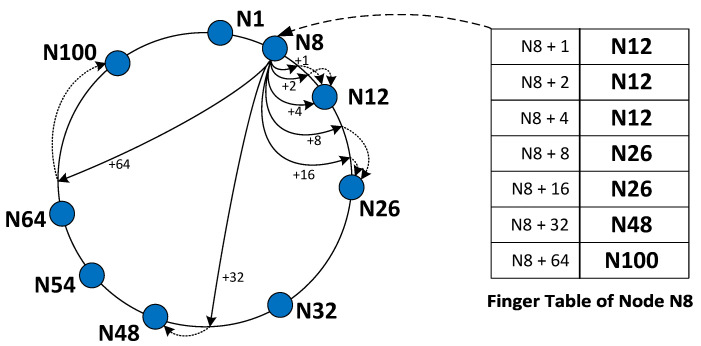
WiCHORD structure and finger table example with m=7 bits; figure inspired from [12].

The WiCHORD protocol as it was originally proposed in [12] is an adaptation of the Chord protocol for P2P systems that was proposed in [9], and therefore some modifications were performed in the structure and operations of the original Chord, in order to bring Chord-like functionality to a wireless sensor network. First of all, the WiCHORD Protocol has the same ring-shaped fixed hash space with *m*-bit addresses and $2^{m}$ total hash keys as Chord, but the difference is that WiCHORD stores only nodes on the hash space and no keys, because it is utilized as a routing protocol for locating nodes within wireless sensor networks [12]. Each node on the hash space of the WiCHORD protocol represents a sensor node device in the physical world, the unique identifier (node ID) of this node in the hash space is obtained by hashing the unique MAC Address of this node device with the SHA-1 algorithm, and this node only maintains contact with O(m) other nodes from the network, which are also known as “fingers” and stored within the node’s finger table [12]. On top of that, WiCHORD follows the basic principle of Chord from [9], which indicates a hash key to be mapped on the next available node on the ring hash space [12]. The structure of a WiCHORD network, which is similar to the structure of the original Chord, is shown in Figure 2, presenting an example network with the finger table of one node, with m=7 bits.

Regarding the operations of the WiCHORD Protocol, in comparison to the original Chord, the following alterations were made to adjust it in order to be suitable for a wireless sensor network use-case scenario. According to [12], the lookup query functionality of WiCHORD retrieves the corresponding node for a requested key in the same way as the original Chord. However, the only valid input for a lookup query in WiCHORD is the MAC address of another node within the network, for data routing and communication between the nodes on the sensor network [12]. Additionally, the functionality of the “Join” operation on WiCHORD updates a few other nodes on the network that will be affected by the new node addition, with a logarithmic complexity similar to Chord, while in contrast to the original Chord, no new keys are transferred to the new node due to the absence of keys on the WiCHORD’s hash space [12]. On the other hand, for sensor node withdrawal cases, a new “Leave” operation has been implemented on WiCHORD to update the contact lists of the affected nodes with a logarithmic complexity in the same way as the “Join” operation functions [12]. In addition, for new WiCHORD sensor network’s build purposes, a new “Build” operation has been implemented in WiCHORD, which calls the “Join” operation for each new node requesting to join the network [12].

### 3.2. Machine-to-Machine Communication Context on the WiCHORD Protocol

A fundamental requirement of the WiCHORD protocol functionality is the concept of internode communication on a wireless sensor network in the physical world. In order to support the different node cooperation and data exchange within the WiCHORD overlay on a wireless sensor network, along with the distinctive operations that this protocol supports, a framework of sensor node communication with data packets and messages has to be defined and implemented. This falls into the concept of machine-to-machine communication (M2M), in which different machines communicate with each other without any human interference [84,85]. The concept of machine-to-machine communication is ideal for a wireless sensor network environment, in which many sensor node devices exchange data packets utilizing wireless communication technologies.

Machine-to-machine (M2M) communication in the WiCHORD protocol applied on a wireless sensor network falls into two major categories. On the one hand, each sensor node device collects data from its onboard sensors and relays them to the gateway node of the network in order for them to be accessible to the end user over the Internet via a cloud computing solution. On the other hand, all the sensor node devices on the network communicate to execute all the different operations that the WiCHORD protocol includes. For instance, each sensor node relays its sensor readings to the gateway node periodically. At the same time, in order to resolve a lookup query for a requested sensor node on a WiCHORD wireless sensor network, the query message originating from the gateway sensor node is forwarded to the corresponding nodes within the WiCHORD network overlay until it reaches the requested node, and this node returns its sensor readings to the gateway. This process involves more than one sensor node that needs to communicate with other nodes and exchange different types of messages. Also, the topology of the wireless sensor network is crucial for successful node device communication.

Therefore, in the following subsections, the required context of machine-to-machine (M2M) communication and the network topology of a wireless sensor network running WiCHORD are defined.

#### 3.2.1. WiCHORD Messages and Their Importance

WiCHORD messages are a core component of the WiCHORD protocol, serving as the primary means of communication within the network. These messages are essential for several reasons:

- **Data transmission:** WiCHORD messages carry sensor data collected by individual nodes to the gateway node. This information can include various environmental readings crucial for smart agriculture decision-making.
- **Network health:** The protocol uses messages to monitor the health and connectivity of the network. Nodes periodically send status updates, ensuring the network is functioning optimally, and that data paths are clear.
- **Scalability and maintenance:** Messages allow the network to scale dynamically. When new nodes are added or removed, messages help update the network’s topology, ensuring efficient data routing.

Within the protocol, WiCHORD messages function to maintain the network’s distributed structure, enabling each node to discover and communicate with other nodes using a distributed hash table (DHT), which is part of the Chord protocol architecture. Each message contains information about the sender node, the intended recipient, query operation directives, and the data payload. This structure facilitates the key operations of the protocol, such as node join/leave, lookup services, and data retrieval, ensuring the protocol’s efficiency and reliability in smart agriculture applications.

#### 3.2.2. WiCHORD Data Packets Architecture

The sensor node devices on a WiCHORD wireless sensor network are utilizing LoRa wireless communication to communicate with each other. This means that they exchange LoRa packets for machine-to-machine (M2M) communication purposes. Therefore, the WiCHORD data packets exchanged between the sensor nodes on a wireless sensor network running the WiCHORD protocol overlay are nested within the payload of a LoRa packet. The maximum quantity of data that a LoRa packet can carry within its payload is 256 bytes, depending on the spreading factor, the coding rate, and the bandwidth [86]. Consequently, the different types and the structure of a WiCHORD LoRa packet are defined for proper internode communication on the sensor network carrying out the operations supported by the WiCHORD protocol overlay. All the different WiCHORD packet types are summarized in Table 3.

The major different cases in which WiCHORD LoRa packets are exchanged between the sensor nodes during the operation of a WiCHORD wireless sensor network are the following. First, when a sensor node has to relay its gathered sensor data back to the gateway sensor node, a “Sensor Data Relay” packet is sent from this sensor node towards the gateway node. This packet contains the hashed node IDs on the WiCHORD hash space of the sender and receiver sensor nodes alongside a payload of the sensor readings.

In case a sensor node device is intended to join or leave the wireless sensor network, a subset of pre-existing sensor network nodes on the WiCHORD overlay have to be updated. Therefore, a “Node Join Request” or “Node Leave Request” packet is sent from that sensor node towards the gateway sensor node, in order for the request to be processed and the WiCHORD network to be updated. This packet contains the unique node ID of the node that sent it alongside the unique identifier of the sensor network that it belongs to. Also, when a sensor node joins or leaves the network, packets with messages to update the successor/predecessor node pointers and finger table are sent from that node toward all the other nodes of the network, but only the nodes that are affected by this procedure respond to that message and update their contact lists. This is because Chord, on which WiCHORD is based, updates only O(logN) other nodes when a new node joins the network of *N* total nodes [9]. Additionally, the “Leave” operation introduced in the WiCHORD protocol has a similar functionality to the “Join” operation of the original Chord protocol, and thus also updates only O(logN) other nodes [12].

A very important operation of the WiCHORD protocol is the sensor node lookup query, in which a sensor node is located within the wireless sensor network via the WiCHORD protocol overlay, and its sensor readings are relayed back to the node that executed the query, given the unique MAC address of the requested sensor node. To perform the sensor node lookup query, the gateway sensor node sends a “Lookup Request” WiCHORD packet, which is forwarded between some of the nodes of the network until it reaches the requested destination node. This packet contains the unique node IDs of the sender and the receiver node along with the node ID of the requested destination node and other useful information as payload. The receiver node ID for the lookup query packet is the ID of each intermediate sensor node to which this packet is forwarded before it reaches the destination.

**Figure 3:**
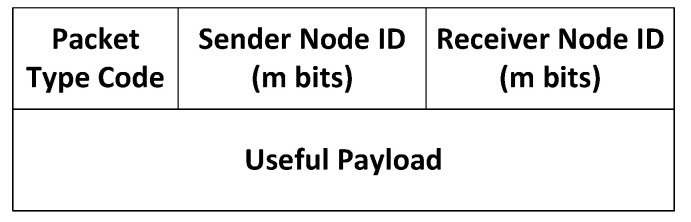
WiCHORD packet architecture.

The structure of a WiCHORD data packet consists of some important attributes such as the query type, the unique node ID of the sender and the receiver sensor node, and the payload of the packet. The sender and receiver node IDs have a size of *m* bits each, with *m* being the maximum number of bits for a key on the WiCHORD hash space. The useful payload varies between the different packet types. For example, the payload of a “Sensor Data Relay” WiCHORD packet contains the sensor readings of the sensor node that sent this packet. The WiCHORD protocol overlay is suitable for LoRa wireless sensor networks, and therefore, the WiCHORD packets are contained within the payload of a LoRa packet. By design, each WiCHORD packet is a string of text encoded in JSON format, with all the packet attributes as key–value pairs. The architecture of a WiCHORD packet is shown in Figure 3.

#### 3.2.3. WiCHORD Wireless Sensor Network Hybrid Topology

**Figure 4:**
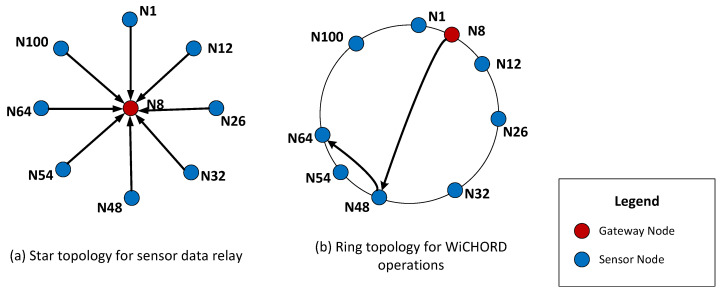
WiCHORD hybrid topology in the case of sensor data relay and while running a WiCHORD lookup query.

As stated above, a wireless sensor network with the WiCHORD protocol overlay has a hybrid functionality either with a direct relay of sensor readings from a node towards the gateway or by running WiCHORD queries on the network. As a result, the topology of a wireless sensor network based on the WiCHORD protocol is hybrid as well. On the one hand, when a sensor node sends its sensor data readings to the gateway sensor node of the LoRa wireless sensor network, the network topology follows the “star” network topology, in which all the simple sensor nodes send data packets to a single gateway node of the network [87,88]. On the other hand, when running WiCHORD queries, the network topology follows the “ring” network topology from the WiCHORD protocol, which is based on the Chord protocol from [9]. This topology is virtual and completely detached from the physical topology of the network in the real world. This is because the nodes are organized on a circular hash space based on their unique node IDs. For example, if two nodes are adjacent in the WiCHORD hash space, they may not be in proximity to the physical network. An example of the hybrid network topology on a wireless sensor network running the WiCHORD protocol is shown in Figure 4.

### 3.3. WiCHORD Optimization and Benefits for Wireless Sensor Networks

The adaptation of the Chord protocol on a wireless sensor network application can bring a variety of benefits. Some notable benefits are, among others, the stability of the network in continuous sensor node connections and disconnections, the total network energy efficiency while running the WiCHORD queries, and the balanced distribution of keys in the nodes of the network. In the following subsections, the benefits of energy efficiency and balanced key distribution are investigated.

#### 3.3.1. Energy Efficiency on a WiCHORD WSN

The energy efficiency in the wireless sensor network is important since the sensor node devices rely on batteries for power. The energy consumption of the WiCHORD protocol is influenced by the total number of sensor node devices that participate in the resolution of a join, leave, or lookup query. Each individual node consumes a constant amount of energy while it participates in a query resolution process. Specifically, in the case of a sensor node device joining or leaving the network, a subset of other sensor node devices have to update their contact details to maintain the stability of the sensor network. This leads to energy consumption since the sensor node device has to use its own MCU to process this join or leave operation. Also, in the case of a sensor node lookup query, the sensor node devices involved in resolving the query have to forward the lookup message to some of the other nodes of the network, until the destination sensor node device is reached. This message-forwarding communication is performed using the sensor node device’s LoRa wireless communication module and therefore energy is consumed. By the design of the Chord protocol from [9], in which the WiCHORD overlay is based on, only a subset of nodes are involved when running a query. The energy efficiency of the “Join”, “Leave”, and “Lookup” operations of WiCHORD on a wireless sensor network using LoRa is discussed in this subsection.

When a new sensor node device intends to join a WiCHORD wireless sensor network, the join operation of the WiCHORD is executed. Since the join operation of the WiCHORD protocol is based on the corresponding join operation of the original Chord that was described in [9], in each join query, only a subset of the total nodes in the network is involved in order to execute the query and update the nodes’ contact details to point to the new node. According to the original Chord from [9], each time a join query is performed, O(logN) total nodes update their list of contacts to include the new node, with *N* being the total number of nodes in the network. The same applies to the WiCHORD protocol as described in [12], because it is an adaptation of Chord for wireless sensor networks.

In case a sensor node device about to leave the network due to insufficient battery, the “Leave” operation was introduced in the WiCHORD as described in [12]. Since the leave operation of the WiCHORD updates the affected nodes in a similar way to the join operation described above, O(logN) total nodes are updated in order to remove the leaving node from their contact lists, in a sensor network of *N* total nodes.

As shown in [12], when performing a lookup query to locate a requested sensor node on a LoRa wireless sensor network running the WiCHORD overlay, a subset of O(logN) nodes are involved to forward the query to the requested node. This is a consequence of the logarithmic complexity of the lookup operation on the original Chord protocol as stated in [9].

As a result, the energy consumption of join, leave, and lookup queries has a logarithmic variation over the total number of nodes in the network, since the number of involved nodes in such queries is a logarithmic function of the total nodes in the network. This leads to a reduction in the total network energy consumption since only a smaller subset of sensor node devices participate in the join, leave, and lookup operations, in comparison to a simple LoRa wireless sensor network, in which the total number of sensor node devices would have to participate in resolving a query.

#### 3.3.2. Storage Efficiency on WiCHORD Sensor Node Devices

On a wireless sensor network application, sensor node devices are based on microcontroller units (MCU) that have a limited amount of onboard memory. In a simple sensor network scenario without the WiCHORD protocol overlay, all the nodes would have to maintain contact lists containing all the node details of the network, in order to support exact match queries for specific nodes of the network. Therefore, when scaling up a wireless sensor network by adding more node devices in the network, the memory of each node would be exceeded. Since the WiCHORD protocol, which is based on the Chord protocol from [9], stores only a few node IDs on each node of the network, as described in [12] and in Figure 2, it can be more efficient in terms of storage efficiency in the onboard memory of each node in the network. In the sensor network overlay of this solution, each node stores O(m) IDs of other nodes in the network as stated in [9,12], with *m* being the maximum number of bits for the keys that belong to the WiCHORD hash space.

### 3.4. Proposed Use-Case Example of the WiCHORD Protocol

**Figure 5:**
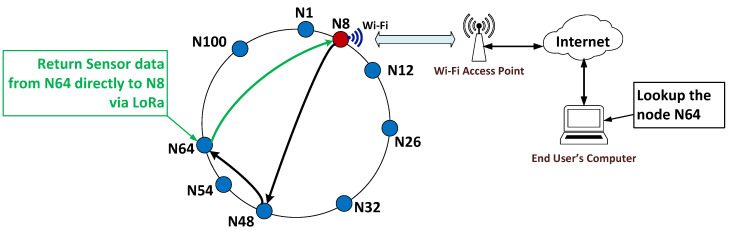
WiCHORD lookup query example on a LoRa WSN.

In this subsection, an example use-case scenario of WiCHORD protocol utilization on a wireless sensor network is presented. First of all, an already deployed LoRa wireless sensor network suitable for the WiCHORD protocol is assumed with some pre-existing sensor nodes already joined in the WiCHORD network. Also, it is assumed that this instance of WiCHORD wireless sensor network is connected with a cloud computing infrastructure from which the end user manages this sensor network. In this use-case scenario, a sensor node lookup query is performed via WiCHORD in order to locate a sensor node within the sensor network. First of all, the user who performs the query provides the unique MAC Address of the requested sensor node. Then, the gateway node of the network receives the lookup query over the Internet and forwards the corresponding WiCHORD lookup packet to the nodes of the WiCHORD wireless sensor network, utilizing the routing algorithm of the Chord protocol from [9] that WiCHORD also uses. When the Lookup message reaches the requested sensor node, this sensor node forwards its most recent sensor readings back to the gateway node directly via LoRa as described in [12]. This use-case scenario is presented in Figure 5.

## 4. Case Study

In this section of this work, a case study for the above proposed WiCHORD protocol overlay for sensor networks on an applications ecosystem for smart agriculture is presented. This applications ecosystem consists of various applications that work together in order to achieve precision agriculture purposes. The different applications of this ecosystem are, among others, a LoRa wireless sensor network suitable for the WiCHORD protocol optimized for a vineyard environment, a cloud computing infrastructure as a service (IaaS) that works as a backend as a service (BaaS) provided by Google Firebase, some machine learning (ML) models that predict plant diseases on vineyards, and a Web dashboard for wireless sensor network management.

**Figure 6:**
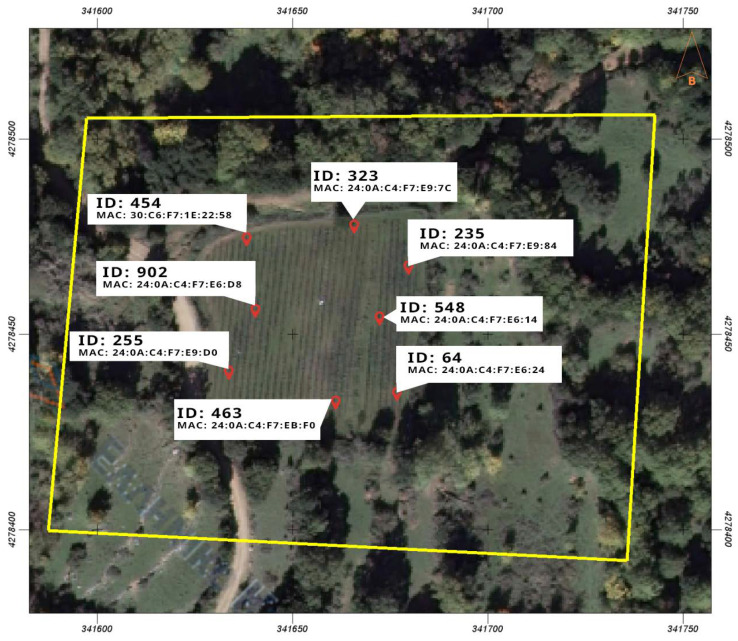
Map view of the wireless sensor network deployment on the vineyard in Koniakos village, Phocis region, Central Greece.

This application ecosystem was named “VineLink Monitoring” and was deployed and tested for the purposes of this study in one vineyard with a total area of 0.4 hectares, situated on the slopes of the “Vardousia” mountain range, near the village of “Koniakos” in the region of “Phocis” in Central Greece, during the Spring and Summer of 2022. A satellite view of the vineyard on which this application was tested, along with the map of the implemented and deployed sensor network on that vineyard is presented in Figure 6. See more details in Appendix A.1.

All these different applications that compile this precision agriculture ecosystem are briefly presented in the following subsections.

### 4.1. VineLink Monitoring

The VineLink Monitoring case study provides a tangible example of the WiCHORD protocol being applied in sensor networks for a vineyard monitoring scenario that was performed on the above-mentioned farm situated near the village of “Koniakos”, in the “Phocis” region of Central Greece (Figure 6), demonstrating real-world benefits and operational effectiveness. The real-world benefits are:

- VineLink Monitoring enables precision viticulture, allowing vintners to understand and respond to the microvariations within their vineyards.
- This system optimizes resource usage—water, nutrients, and treatments are applied judiciously, leading to significant savings and environmental benefits.
- The monitoring system excels in early pest and disease detection, which is crucial in maintaining vine health and reducing crop loss.
- It also streamlines operations, as the automated data collection frees up labor from in-person vineyard monitoring for other essential agricultural activities and provides the farmers with a real-time overview of their field.

The operational functionality elements are:

- In the field, VineLink Monitoring deploys a network of sensor nodes strategically placed around the vineyard, based on the ideal model of sensor network for the WiCHORD protocol as described on Section 3.
- These sensors gather critical data points, including climatic conditions and soil moisture levels.
- The gateway node aggregates these data and communicates them efficiently via the WiCHORD protocol, ensuring prompt and reliable data delivery to the central management system.
- The resultant analysis leads to informed decisions, optimizing vineyard practices such as irrigation timing, targeted fertilization, and effective plant disease treatments.

Through VineLink Monitoring, vineyards have witnessed improved grape quality and yield consistency. The system has proven its value in enhancing sustainability and economic viability, showcasing how advanced IoT applications like WiCHORD can revolutionize traditional agricultural practices.

### 4.2. Wireless Sensor Network

**Figure 7:**
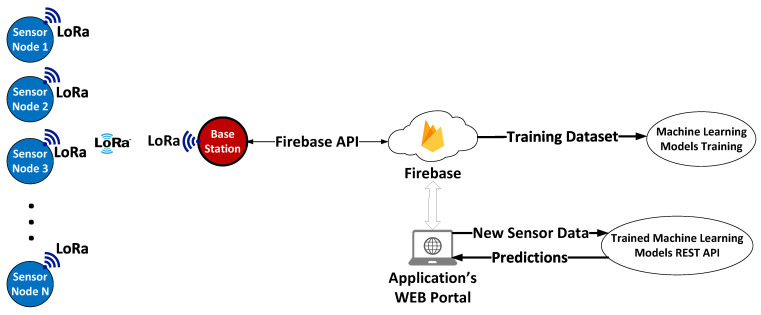
The wireless sensor network architecture, as a part of this case study.

**Figure 8:**
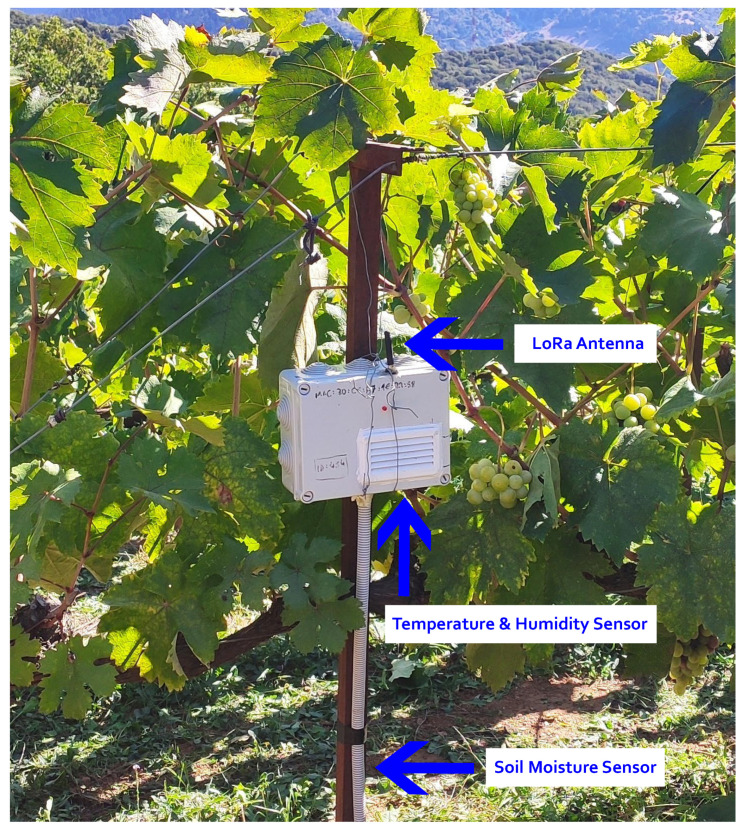
The implemented sensor node device deployed on the vineyard.

**Figure 9:**
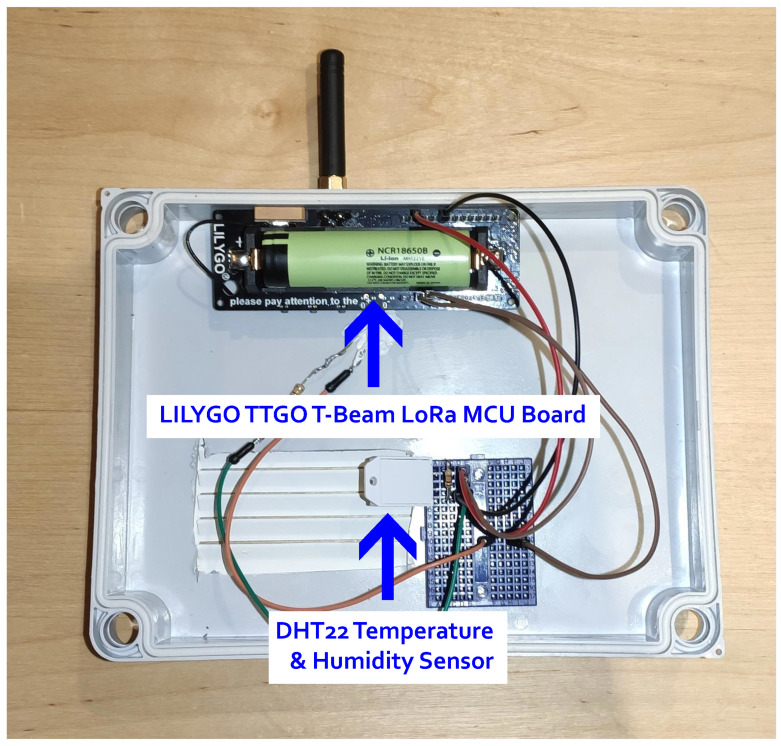
The inner workings of the implemented sensor node device.

In order to collect useful metrics regarding plant disease forecasting in a vineyard environment for this precision agriculture application, a wireless sensor network based on the LoRa wireless communication technology was built. In this wireless sensor network, the WiCHORD protocol, proposed initially in [12] and also described in Section 3 of this work, was applied as an overlay, and all the node devices in the network were assigned with a unique node ID resulting from SHA-1 hashing on the node’s unique MAC Address. This wireless sensor network was comprised of some simple sensor nodes and one gateway node. Each sensor node consisted of an ESP32 microcontroller unit (MCU) with a LoRa wireless communication module embedded on the LILYGO TTGO T-Beam LoRa MCU board, connected with some sensors for local conditions’ monitoring. The sensors on each sensor node monitored the temperature, humidity, and soil moisture of the local environment in which this sensor node was deployed. To monitor these local conditions, the DHT22 temperature and humidity sensor was integrated into the sensor node devices, while for the soil moisture, the sensor node device used a simple off-the-shelf capacitive soil moisture sensor. Every sensor node device relayed its sensor readings as WiCHORD LoRa packets to the gateway node of the network periodically. To connect this wireless sensor network with the other applications of the smart agriculture ecosystem, the gateway node utilized an API provided by the Google Firebase platform, in order to post the received sensor readings to the application’s database that was hosted in Firebase. The vineyard on which this network was deployed is divided into regions, and each sensor node device was assigned to one specific region. The map of the deployment of this WSN on the vineyard is shown in Figure 6, while the architecture of this LoRa wireless sensor network and how it is connected with the rest of the ecosystem is presented in Figure 7. A picture of the implemented sensor node device for this case study, as it was deployed on the vineyard, is presented in Figure 8. The inner workings of this device are shown in Figure 9.

### 4.3. Applications’ Cloud Infrastructure

The backend infrastructure of this application ecosystem was built using cloud computing technologies provided by the Google Firebase platform. Google Firebase is a backend as a service (BaaS) providing services such as databases, user authentication, and web hosting for such applications. This precision agriculture ecosystem utilized a NoSQL document database provided by Firebase, in order to store the received data from the sensor nodes on a JSON format in real time. Also, the Web dashboard application of this ecosystem was hosted on Firebase Hosting and the user authentication in all of the components of this ecosystem was managed by Firebase Authentication.

### 4.4. Plant Disease Forecasting with Machine Learning Techniques Using Sensor Data

The sensor data collected from the above-mentioned LoRa wireless sensor network were utilized in order to train some machine learning (ML) models for plant disease forecasting in vineyards. One of the very common plant diseases in vineyards, which is mentioned also in Table 1, is grape downy mildew (GDM), which affects grapes in a negative way and causes damage to the quality and the quantity of the vineyard agricultural produce. Some of the major factors affecting GDM are the local temperature and humidity along with the rainfall in the area of the affected vine [70,71,72,73]. Therefore, the above-mentioned wireless sensor network (Figure 6, Figure 7, Figure 8 and Figure 9) measured the local temperature and humidity of the vines, using the DHT22 temperature and humidity sensor (Figure 9), and these data were used to train the machine learning models that were implemented. In this project, the data used to train the following machine learning models were collected from the aforementioned vineyard in Koniakos in Central Greece, which is shown in Figure 6, during the Spring and Summer of 2022.

The implemented machine learning models predicted the susceptibility and incubation period of grape downy mildew in the affected vine. Specifically, these machine learning models were trained using the dataset collected by the above-mentioned wireless sensor network. This dataset contained the value pairs of local temperature and humidity for each region of the vineyard as features along with the corresponding labels of grape downy mildew susceptibility (positive/negative) and incubation period (in days) for these value pairs. These labels were assigned for these pairs manually by combining the specifications of the grape downy mildew found in the related agricultural literature [70,71,72,73]. Table 4 shows how susceptibility and incubation period of the GDM plant disease varied depending on the local temperature and humidity of the affected vine plant. Therefore, the temperature and humidity features on the dataset were distributed into the various GDM susceptibility and incubation classes, by following the specification presented on Table 4. In brief, 70% of this dataset was used for the training of the implemented machine learning models, and the rest, 30% of the dataset, was used to evaluate the prediction accuracy of these models.

To forecast grape downy mildew susceptibility which is a binary classification task, a set of well-known classifier models, including a decision tree, a random forest, and a Gaussian naïve Bayes, were implemented with their default parameters and trained with the above-mentioned sensor network dataset, using the Scikit-Learn module [89] in the Python programming language. Also, to forecast the incubation period of grape downy mildew plant disease which is a multiclass classification task, a set of classifiers, including a decision tree, a random forest, and a Gaussian naïve Bayes, was selected to be trained using default parameters and the sensor network dataset, with Scikit-Learn [89].

The prediction accuracy of the above models was evaluated using the accuracy, precision, recall, and f1 scores, by making predictions about the susceptibility and incubation period of GDM using the test dataset’s temperature and humidity input value pairs, with promising combined results. In particular, for both the cases of grape downy mildew susceptibility (positive/negative) and incubation (in days), the initial datasets had less than 100,000 tuples of data, and the label classes were separated well without overlapping. This means that the decision process for this dataset was simple, following the agricultural rules regarding the conditions affecting the grape downy mildew plant disease. This resulted in very high accuracy scores of more than 99% for the decision tree and random forest models in both cases, while the naïve Bayes model scored an accuracy of 89% in the case of grape downy mildew susceptibility and an accuracy of about 99% for the case of grape downy mildew incubation. In contrast to the naïve Bayes, decision tree may be prone to the problem of overfitting, while naïve Bayes may also perform better and avoid overfitting with well-separated datasets like the one in this project. The combined evaluated scores are shown in Table 5.

To integrate these machine learning models with the broader application ecosystem, an API using the Flask web framework was implemented. This API received temperature and humidity data pairs via HTTP requests and returned the machine learning predictions from the above-mentioned models.

### 4.5. Applications’ Web Dashboard

The Web dashboard implemented as part of this smart agriculture application informed the end user about the collected data from the wireless sensor network along with the corresponding predictions from the above-mentioned machine learning models. This Web dashboard application was developed using the React.JS framework in the JavaScript programming language, and the Bootstrap 5 CSS framework for the application’s user interface. The connection with the Google Firebase backend was established using the Firebase Web SDK provided by Google.

## 5. Results

To verify the efficiency of the proposed WiCHORD protocol overlay for wireless sensor networks, a simulator application was developed and some simulation experiments were carried out. First of all, using the Python 3 programming language, the Jupyter Notebook programming environment and libraries such as Pandas [90] and Matplotlib [91], the simulator application that was developed to initially evaluate the WiCHORD Protocol from [12] was extended in order to evaluate some of the WiCHORD functionalities discussed in this work. See more details about the simulator development in Appendix A.2. In the following subsections, the balanced distribution of node IDs in the network nodes, along with the total energy efficiency in the network nodes that the WiCHORD achieved, was evaluated by performing the corresponding simulation experiments.

### 5.1. Sensor Node Storage Efficiency Evaluation

As was mentioned in the methodology section of this work, the WiCHORD protocol overlay maintains contact lists in the nodes of the network for routing purposes. These contact lists are stored within the memory of the nodes, which has limited capacity, and each node stores only a few other node IDs in its contact list, as the WiCHORD protocol dictates in [12]. Therefore, if the sensor network scales up in terms of total nodes population, it is important that each node stores only a subset of node IDs in its memory.

**Figure 10:**
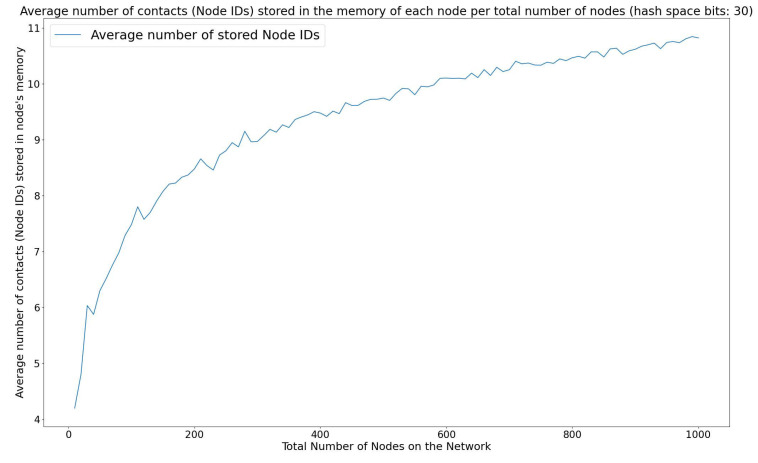
Storage efficiency simulation results.

In this experiment, the average number of node IDs (contacts) that were stored within the memory of the nodes was calculated over the total number of nodes on a WiCHORD sensor network. Specifically, in each iteration of this experiment, random instances of WiCHORD networks of *N* total nodes were created, with N∈[10,1000] incremented by 10 in each experiment iteration. Then, in each iteration of the experiment, the average number of unique node IDs stored in the finger table and the predecessor pointer of each node was calculated for all the nodes of the network. Finally, the combined results of this experiment with the average number of stored contacts in the node’s memory per total number of nodes in the network are shown in Figure 10, where as the number of total network node scales up, the average number of contacts for each node is a small subset of the total nodes in the network.

### 5.2. WiCHORD Operations’ Energy Efficiency Evaluation

Energy efficiency is crucial in a wireless sensor network application in which sensor node devices rely on batteries for power. One of the key motivations for the implementation of the WiCHORD protocol was the fact that WiCHORD “Join”, “Leave”, and “Lookup” operations have a logarithmic complexity, and thus in each operation, only a few of the total nodes of the network are involved. When a node is involved in a “Join” or “Leave” operation, it usually has to exchange messages with the other nodes in the network, in order to update its contact list by including or removing another sensor node. Also, when a node of the network is participating in a “Lookup” query, it basically has to forward the lookup message to the other nodes of the network until the destination node is reached, or if this node is the destination node, then this node has to respond with its sensor data to the node that performed the lookup query. In these cases, some of the nodes on the sensor network running WiCHORD, are using their onboard LoRa module in order to communicate with the other nodes of the network to resolve the corresponding query and this results in energy consumption from the wireless communication along with the MCU of the sensor node device not being idle. Assuming that each “Join”, “Leave”, and “Lookup” operation running on the WiCHORD network involving one node during its execution has a constant energy consumption $c_{join},c_{leave}$, and $c_{lookup}$, respectively, in the following experiments, the average number of involved nodes per total nodes on the network was calculated, and thus the energy consumption of these operations could be calculated as the product of the total involved nodes times the corresponding constant for each operation.

**Figure 11:**
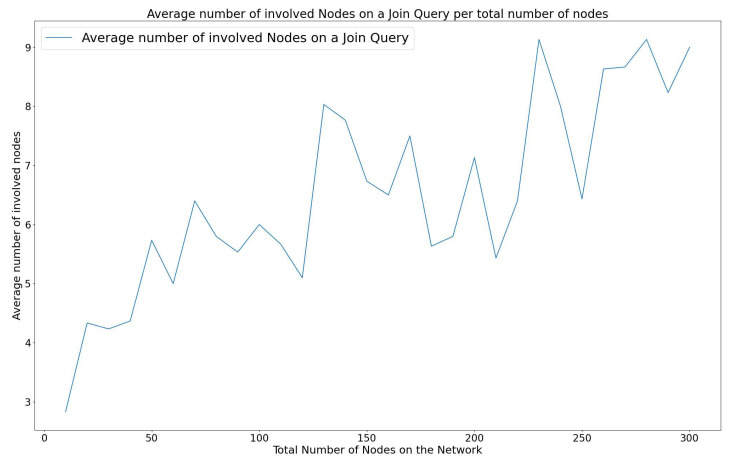
WiCHORD join operation simulation results.

For the join operation of the WiCHORD protocol, the following experiment was conducted: The average number of involved nodes on a join query per total network nodes was calculated. Specifically, for random simulated instances of WiCHORD sensor networks comprised of *N* total nodes, with N∈[10,300] incremented by 10 in each step, a number of *n* node join queries were performed by adding a new node with a random MAC address on the network and calculating the number of unique nodes involved in this query. Each time these queries were performed, the WiCHORD network instance was initialized to keep the total number of nodes the same for all experiments. For each step of *N*, a total of n=30 simulation experiments were performed, and the average total of nodes involved in a join query per total network nodes was calculated for this value of *N*. The combined results of the join operation evaluation are presented in Figure 11. Results from this experiment indicate that in the case of a new sensor node joining the network, only a few other sensor nodes were involved in resolving that request, and therefore, less energy was consumed in the sensor network, in comparison to a network-flooding scenario in which all the network nodes have to update their contact lists.

**Figure 12:**
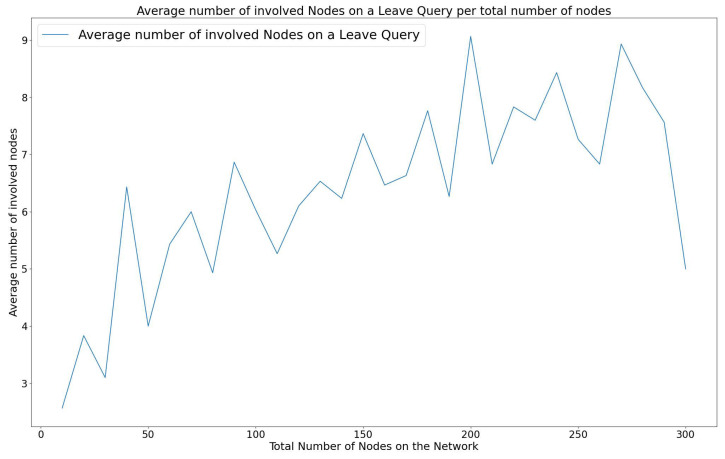
WiCHORD leave operation simulation results.

Similar to the above-mentioned experiment for the evaluation of the WiCHORD join operation, a simulation experiment to evaluate the efficiency of the leave operation of WiCHORD was conducted. This experiment was conducted with the same parameters as the previous one with the only difference being that in each step, a number of leave queries were resolved by selecting a random node of the network to be removed, in order to measure the average number of total nodes updated when a pre-existing node left the network. The combined results from the evaluation of the leave operation are shown in Figure 12.

**Figure 13:**
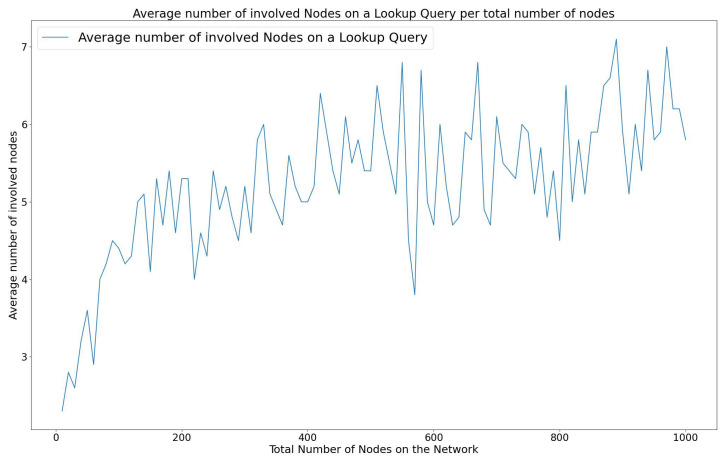
WiCHORD lookup operation simulation results.

To evaluate the lookup operation of WiCHORD in terms of energy efficiency, the average number of nodes participating in resolving a lookup query per total network nodes was measured in the following experiment. For random WiCHORD sensor network instances with a total number of nodes in the range [10,1000] incremented by 10 in each step, 10 random lookup queries were performed for each value of total network nodes, the same way as the lookup-query path-length evaluation experiment from [12], to calculate the average number of involved nodes per total network nodes. The total number of nodes involved in a lookup query was calculated by counting the intermediate sensor nodes that the query was forwarded ti, the destination node, and the origin node. The combined results of the lookup operation evaluation are presented in Figure 13. These results indicate that in a lookup query, the number of involved nodes verified the logarithmic complexity of the WiCHORD Lookup operation, and thus, energy was saved in the sensor network by involving fewer nodes to resolve a query, in comparison to a network-flooding case in which all the sensor nodes would receive the lookup query and then would have to relay their sensor readings back to the gateway node of the sensor network.

### 5.3. Combined Results Implication and Discussion

The above-obtained results from the simulation of the WiCHORD protocol overlay for wireless sensor networks are summarized as follows. The WiCHORD protocol is an overlay that runs on top of wireless sensor networks and utilizes the structure of a distributed hash table to efficiently manage the sensor node contact lists stored within the memory of the sensor node devices and the total energy consumption of a sensor network during the sensor node join, leave, and lookup queries, in comparison with a simple wireless sensor network without an overlay protocol. In a simple wireless sensor network, all the sensor nodes within the network have to maintain contact lists containing the total number of sensor node identifiers and have to update their contact lists when a node joins or leaves the network, and all the sensor nodes have to participate when a sensor node lookup query floods the network. Therefore, by applying the WiCHORD protocol overlay to a wireless sensor network, the above simulation results indicate that each node device maintains only a few sensor node identifiers as contacts. Also, only a logarithmic subset of the total sensor network nodes has to participate in the case of a sensor node’s join, leave, and lookup query. This means that less energy is consumed in the whole sensor network when these queries are performed since fewer sensor node devices are active during these query operations.

## 6. Discussion

The WiCHORD protocol, stemming from the foundation work of the Chord protocol, presents an evolutionary and innovative approach to wireless sensor networks. The resulting inner working design considers the unique challenges and requirements of WSNs, ensuring efficiency and scalability without compromising the integrity of the system. Generally, the following aspects can be discussed:

- Distinct node-centric approach: One of the main aspects of WiCHORD is its internal design focused on the nodes rather than the keys, as in the Chord protocol. By representing each sensor node device in the physical world within the hash space, WiCHORD enables a self-alignment with the nature of WSNs where the device location and communication path are crucial. Therefore, the resulting reduction in system complexity, which is derived from having only nodes and no keys in the hash space, transforms the system into a less resource-intensive mechanism, leading to a valuable protocol for WSNs that can operate under resource constraints.
- Effective lookup mechanism: The lookup operation within WiCHORD, although inspired by Chord, is particularly tailored to WSNs. By relying on the MAC address for data routing, WiCHORD effectively breaks down the narrow space in the search domain, ensuring an efficient communication among nodes. This approach is particularly beneficial for WSNs, which often face high data traffic, ensuring a reduced latency in the network.
- Enhanced join/leave operations: The adaptability of WiCHORD is self-evident in its join and leave operations. The functions not only accommodate the dynamic nature of WSNs, where nodes might frequently join or leave the network, but also maintain the integrity of the network with a logarithmic complexity. Moreover, the omission of key transfers in the join operation serves to further streamline the process, reducing potential bottlenecks in the overall system.

As per the results, the following concluding aspects could be discussed:

- Storage efficiency: We simulated how many keys a node contained in the network, as LoRa is limited in memory and all nodes could not save all keys as this would fill up the memory. WiCHORD, as it only knows the successor and the predecessor, results in a reduction of log(n) in the neighboring nodes, for the unique keys.
- Energy efficiency: we performed a simulation to check the average nodes involved as per the total number of nodes in the network for various numbers of simulation nodes.
  **Join operation:**
  1. Streamlined process: by avoiding the transfer of keys when a new node joins, WiCHORD minimizes unnecessary data transmissions, which would otherwise consume more energy.
  2. Logarithmic complexity: The join operation’s logarithmic nature means that as the network grows, only a logarithmic number of messages are needed for a node to join. This reduces the total number of message transmissions, conserving energy.
  3. Selective node updating: Rather than broadcasting or updating the entire network when a new node joins, only a subset of nodes (those affected by the new node) are updated. This targeted approach conserves energy by reducing unnecessary communication.
  **Leave Operation:**
  1. Targeted node update: Similar to the join operation, when a node leaves the network, only affected nodes need to be updated. By limiting the number of nodes involved in the process, energy consumption due to communication is minimized.
  2. Avoidance of global network reorganization: Instead of reorganizing or recalculating the entire network structure when a node leaves, the leave operation’s efficient design ensures minimal disturbances. This helps in preventing widespread data transmissions, saving energy.
  **Lookup operation:**
  1. MAC address dependency: Relying solely on the MAC address for data routing not only simplifies the lookup process but also reduces the chances of redundant or unnecessary data transmissions. Efficient data routing conserves energy by ensuring that messages take the shortest and most direct path to their destination.
  2. Reduced latency: A well-optimized lookup operation ensures that data reach their destination faster. Quicker data transmission means radios are active for shorter durations, leading to energy conservation.
  3. Minimized traffic: by refining the search domain to the MAC address and utilizing the efficient principles derived from the Chord protocol, the lookup operation minimizes network traffic, reducing energy consumption from frequent data transmissions.

## 7. Conclusions and Future Directions

This study demonstrated the effectiveness of the WiCHORD protocol in enhancing the operational efficiency of wireless sensor networks (WSNs) in smart agriculture, particularly through advanced machine learning techniques and improved sensor node operations. The deployment of machine learning models for grape downy mildew (GDM) forecasting in vineyards showcased a remarkable predictive accuracy. Our models, utilizing sensor data for local temperature and humidity, achieved accuracy scores above 99% for decision tree and random forest classifiers. This significant level of precision illustrates the potential of integrating IoT technologies with data-driven agricultural practices.

In terms of storage efficiency, the WiCHORD protocol proved effective in managing sensor node resources. The experimental results showed that the average number of node IDs stored in sensor nodes remained low, even as the network scaled up. This efficiency is critical in maintaining the viability and performance of large-scale sensor networks, particularly where node devices have limited memory capacity.

Furthermore, our evaluations of the WiCHORD protocol’s energy efficiency during key operations—namely, node join, leave, and lookup processes—confirmed its energy-saving benefits. The protocol’s design, which involved only a subset of nodes in each operation, significantly reduced the overall energy consumption compared to scenarios where all nodes participated. This aspect is crucial in extending the operational life of battery-powered sensor nodes, a key factor for sustainable smart agriculture applications.

However, challenges remain, particularly in adapting the protocol to the unpredictable and varied conditions of agricultural environments. Future work should focus on further optimizing the WiCHORD protocol for complicated environmental conditions and exploring ways to enhance its energy efficiency. Additionally, reinforcing the security measures in data transmission within WSNs will be a crucial aspect of ongoing research.

Looking ahead, the WiCHORD protocol’s adaptability in varied agricultural environments will be a key area of development. Its ability to integrate with complex sensor types and data sources is crucial for meeting the specific needs of different crops and farming conditions. Future enhancements should focus on incorporating adaptive algorithms for more personalized agricultural interventions, aligning with the objectives of precision agriculture. These developments are essential for achieving higher crop yields, environmental sustainability, and economic benefits in the evolving landscape of smart farming.

Additionally, regarding the prediction of plant diseases in vineyards for the VineLink Monitoring case study, further directions of this work include the implementation, training, and evaluation of machine learning models. This includes the incorporation of a field report on the status of GDM plant disease development on the vines as a feature, in alignment with the weather data from the sensors deployed on the field. The plant disease prediction with machine learning trained with sensor data can be extended to other vineyard plant diseases in vineyards as well.

In conclusion, the WiCHORD protocol presents a robust framework for smart agriculture, combining the benefits of machine learning for disease prediction with an efficient sensor network management. Continuous improvements in these areas will create a foundation for more resilient, efficient, and sustainable agricultural practices, utilizing the power of IoT and advanced data analytics.

## Figures and Tables

**Table 1:** Common diseases affecting vineyards, affected by local environmental conditions.

Disease	Description (Local Name)	Factors Affecting	Study Focus	Reference
**Grape downy mildew**	Disease caused by *Plasmopara viticola*. **Symptoms**: yellowish patches on leaves; white mold beneath.	Temperature. Humidity. Wet conditions.	✓	[70,71,72,73]
**Uncinula necator**	Fungal infection causing white spots on leaves, shoots. **Also known as** powdery mildew.	Dry conditions. Warm temperatures.	x	[74]
**Black rot**	Caused by *Guignardia bidwellii*. **Affects** all green vine parts leading to necrotic lesions.	High humidity. Rainfall.	x	[75]
**White rot**	Fungal infection by *Coniella diplodiella* leading to rot.	Extended wetness. Plant injuries	x	[76]
**Eutypa dieback**	Wood decay from *Eutypa lata*. **Symptoms**: stunted growth, “tiger-stripe” leaves.	Pruning wounds. Rain.	x	[77]
**Botryosphaeria dieback**	Disease from *Botryosphaeria* genus. **Results in** cankers, dieback of shoots.	Pruning wounds. Warm temperatures.	x	[77,78]
**Botrytis bunch rot**	From *Botrytis cinerea*. **Affects** berries, especially in tight-clustered grape varieties.	High humidity. Tight grape clusters.	x	[79,80]

**Table 2:** SOTA comparison of research studies with emphasis on WSN technologies and Chord-based applications.

Refs.	Main Focus	Technology	WSN Technology	Protocol	Chord-Based
[81]	Communication protocols, energy-efficiency	Various	LoRa	Multiple	No
[82]	IoT system performance	IoT	LoRa	Multiple	No
[28]	Supply chain management in agriculture	P2P WSNs	LoRa	P2P	No
[20]	Data delivery	WSNs	Typical	Chord	Yes
[21]	Routing protocol	WSNs	Typical	Chord	Yes
[83]	Dynamic rotation and query efficiency	WSNs	Typical	Chord	Yes
[12]	Protocol application to the Internet of things	P2P WSNs	LoRa	Chord	Yes
WiCHORD+	Protocol ecosystem in the Internet of things	P2P WSNs	LoRa	Chord	Yes

**Table 3:** Summary of the different WiCHORD packet types.

Packet Type	Description
Sensor_Data_Relay	Relays a node’s current sensor data to the gateway node. Sent from a sensor node towards the gateway.
Node_Join_Request	Represents the “hello” request of a new sensor node to join an already running pre-existing WiCHORD wireless sensor network.
Node_Leave_Request	Represents the “leave” request of an existing sensor node to leave the WiCHORD wireless sensor network that it belongs to.
Lookup_Request	Sensor node lookup request that is forwarded between the nodes of the sensor network and towards the requested sensor node.

**Table 4:** Temperature and Humidity factors affecting GDM plant disease. Table compiled with data combined from [70,71,72,73].

Temperature	Humidity	GDM Susceptibility	GDM Incubation Period
13–16 ${}^{\circ }$C	≥85%	Positive	8–9 days
16–20 ${}^{\circ }$C	≥85%	Positive	7 days
20–28 ${}^{\circ }$C	≥85%	Positive	5–6 days
≤13 ${}^{\circ }$C and ≥28 ${}^{\circ }$C	≤85	Negative	No incubation

**Table 5:** Combined machine learning classification models evaluation scores.

Use Case	Classifier	Accuracy Score	Precision Score	Recall Score	F1 Score
GDM Susceptibility	Decision tree	99.99%	0.99	0.99	0.99
GDM Susceptibility	Random forest	99.99%	0.99	0.99	0.99
GDM Susceptibility	Gaussian naïve Bayes	89.81%	0.86	0.95	0.90
GDM Incubation	Decision tree	99.99%	0.99	0.99	0.99
GDM Incubation	Random forest	99.99%	0.99	0.99	0.99
GDM Incubation	Gaussian naïve Bayes	96.85%	0.96	0.96	0.96

## Data Availability

The data presented in this study are available on request from the corresponding author.

## Abbreviations

The following abbreviations are used in this manuscript:

IoT	Internet of things
AI	Artificial intelligence
P2P	Peer-to-peer systems
DHT	Distributed hash tables
WSNs	Wireless sensor networks
MCU	Microcontroller unit
LPWAN	Low-power wide-area network
LoRa	Long range
PA	Precision agriculture
ML	Machine learning
UAVs	Unmanned aerial vehicles
MOT	Multiple object tracking
TSP	Traveling salesman problem
UAS	Unmanned aerial systems
PPP	Plant protection products
GTDs	Grapevine trunk diseases
M2M	Machine-to-machine
MAC address	Media access control address
JSON	Javascript object notation
IaaS	Infrastructure as a service
BaaS	Backend as a service
RSSI	Received signal strength indicators
SNR	Signal-to-noise ratios
GDM	Grape downy mildew

## Appendix A

### Appendix A.1

The vineyard on which the case study of this work from Section 4 was tested, provided by the Company: “Balatsouras Winery”.

### Appendix A.2

The code of the presented simulator of WiCHORD+ can be found on GitHub, at the following URL: https://github.com/takis104/WiCHORD-chord-application-P2P-Wireless-Sensor-Network-IoT.

## References

[1] Navarro E., Costa N., Pereira A. (2020). A Systematic Review of IoT Solutions for Smart Farming. Sensors.

[2] Rehman A., Saba T., Kashif M., Fati S.M., Bahaj S.A., Chaudhry H. (2022). A revisit of internet of things technologies for monitoring and control strategies in smart agriculture. Agronomy.

[3] Rose K., Eldridge S., Chapin L. (2015). The internet of things: An overview. Internet Soc. (ISOC).

[4] Nauman A., Qadri Y.A., Amjad M., Zikria Y.B., Afzal M.K., Kim S.W. (2020). Multimedia Internet of Things: A comprehensive survey. IEEE Access.

[5] Kandris D., Nakas C., Vomvas D., Koulouras G. (2020). Applications of wireless sensor networks: An up-to-date survey. Appl. Syst. Innov..

[6] Kim B.S., Kim K.I., Shah B., Chow F., Kim K.H. (2019). Wireless sensor networks for big data systems. Sensors.

[7] Bahashwan A.A., Anbar M., Abdullah N., Al-Hadhrami T., Hanshi S.M. (2021). Review on common IoT communication technologies for both long-range network (LPWAN) and short-range network. Advances on Smart and Soft Computing: Proceedings of ICACIn 2020.

[8] Raja Basha A. (2022). A review on wireless sensor networks: Routing. Wirel. Pers. Commun..

[9] Stoica I., Morris R., Liben-Nowell D., Karger D., Kaashoek M., Dabek F., Balakrishnan H. (2003). Chord: A scalable peer-to-peer lookup protocol for Internet applications. IEEE/ACM Trans. Netw..

[10] Stoica I., Morris R., Karger D., Kaashoek M.F., Balakrishnan H. (2001). Chord: A scalable peer-to-peer lookup service for internet applications. ACM SIGCOMM Comput. Commun. Rev..

[11] Zave P. (2012). Using lightweight modeling to understand Chord. ACM SIGCOMM Comput. Commun. Rev..

[12] Balatsouras C.P., Karras A., Karras C., Tsolis D., Sioutas S. WiCHORD: A Chord Protocol Application on P2P LoRa Wireless Sensor Networks. Proceedings of the 2022 13th International Conference on Information, Intelligence, Systems & Applications (IISA).

[13] Galuba W., Girdzijauskas S., Liu L., Özsu M.T. (2009). Peer-to-Peer System. Encyclopedia of Database Systems.

[14] Androutsellis-Theotokis S., Spinellis D. (2004). A Survey of Peer-to-Peer Content Distribution Technologies. ACM Comput. Surv..

[15] Karger D.R., Ruhl M. Simple Efficient Load Balancing Algorithms for Peer-to-Peer Systems. Proceedings of the Sixteenth Annual ACM Symposium on Parallelism in Algorithms and Architectures.

[16] Zhang H., Wen Y., Xie H., Yu N. (2013). Distributed Hash Table: Theory, Platforms, and Applications.

[17] Rowstron A., Druschel P. (2001). Pastry: Scalable, decentralized object location, and routing for large-scale peer-to-peer systems. Proceedings of the Middleware 2001: IFIP/ACM International Conference on Distributed Systems Platforms.

[18] Maymounkov P., Mazieres D. (2002). Kademlia: A peer-to-peer information system based on the xor metric. Peer-to-Peer Systems: Proceedings of the First InternationalWorkshop, IPTPS 2002 Cambridge, MA, USA, 7–8 March 2002.

[19] Fersi G., Louati W., Ben Jemaa M. (2013). Distributed Hash table-based routing and data management in wireless sensor networks: A survey. Wirel. Netw..

[20] Cheklat L., Amad M., Omar M., Boukerram A. Energy Efficient Physical Proximity based Chord Protocol for Data Delivery in WSNs. Proceedings of the 2018 International Conference on Applied Smart Systems (ICASS).

[21] Cheklat L., Amad M., Omar M., Boukerram A. (2021). Chearp: Chord-based hierarchical energy-aware routing protocol for wireless sensor networks. Comput. Sci. Inf. Syst..

[22] Ali M., Langendoen K. A case for peer-to-peer network overlays in sensor networks. Proceedings of the International Workshop on Wireless Sensor Network Architecture (WWSNA’07).

[23] Awad A., Sommer C., German R., Dressler F. Virtual Cord Protocol (VCP): A flexible DHT-like routing service for sensor networks. Proceedings of the 2008 5th IEEE International Conference on Mobile Ad Hoc and Sensor Systems.

[24] Guidara A., Fersi G., Derbel F. Lookup service for fog-based indoor localization platforms using chord protocol. Proceedings of the 2020 International Wireless Communications and Mobile Computing (IWCMC).

[25] Carbajo R.S., Mc Goldrick C. (2017). Decentralised peer-to-peer data dissemination in wireless sensor networks. Pervasive Mob. Comput..

[26] Sioutas S., Tsoumakos D., Panaretos A., Tzimas G., Karydis I., Tsolis D. (2012). SART: Speeding up query processing in sensor networks with an autonomous range tree structure. ACM SIGAPP Appl. Comput. Rev..

[27] Sioutas S., Triantafillou P., Papaloukopoulos G., Sakkopoulos E., Tsichlas K., Manolopoulos Y. (2013). ART: Sub-logarithmic decentralized range query processing with probabilistic guarantees. Distrib. Parallel Databases.

[28] Karras A., Karras C., Drakopoulos G., Tsolis D., Mylonas P., Sioutas S., Maglogiannis I., Iliadis L., Macintyre J., Cortez P. (2022). SAF: A Peer to Peer IoT LoRa System for Smart Supply Chain in Agriculture. Artificial Intelligence Applications and Innovations.

[29] Preethi C., Brintha N., Yogesh C. (2021). An Comprehensive Survey on Applications of Precision Agriculture in the Context of Weed Classification, Leave Disease Detection, Yield Prediction and UAV Image Analysis. Adv. Parallel Comput. Technol. Appl..

[30] Aslan M.F., Durdu A., Sabanci K., Ropelewska E., Gültekin S.S. (2022). A Comprehensive Survey of the Recent Studies with UAV for Precision Agriculture in Open Fields and Greenhouses. Appl. Sci..

[31] Singh R.K., Aernouts M., De Meyer M., Weyn M., Berkvens R. (2020). Leveraging LoRaWAN Technology for Precision Agriculture in Greenhouses. Sensors.

[32] Goel S., Guleria K., Panda S.N. (2022). Machine learning techniques for precision agriculture using wireless sensor networks. ECS Trans..

[33] Rodríguez S., Gualotuña T., Grilo C. (2017). A system for the monitoring and predicting of data in precision agriculture in a rose greenhouse based on wireless sensor networks. Procedia Comput. Sci..

[34] Lloret J. Edge Computing in Precision agriculture. Proceedings of the 2022 Seventh International Conference on Fog and Mobile Edge Computing (FMEC).

[35] Schizas N., Karras A., Karras C., Sioutas S. (2022). TinyML for Ultra-Low Power AI and Large Scale IoT Deployments: A Systematic Review. Future Internet.

[36] Karras A., Karras C., Giotopoulos K.C., Tsolis D., Oikonomou K., Sioutas S. (2023). Federated Edge Intelligence and Edge Caching Mechanisms. Information.

[37] Rahmouni M., Hanifi M., Savaglio C., Fortino G., Ghogho M. An AIoT Framework for Precision Agriculture. Proceedings of the 2022 IEEE Intl Conf on Dependable, Autonomic and Secure Computing, Intl Conf on Pervasive Intelligence and Computing, Intl Conf on Cloud and Big Data Computing, Intl Conf on Cyber Science and Technology Congress (DASC/PiCom/CBDCom/CyberSciTech).

[38] Singh R.K., Berkvens R., Weyn M. (2021). AgriFusion: An Architecture for IoT and Emerging Technologies Based on a Precision Agriculture Survey. IEEE Access.

[39] Liu J., Xiang J., Jin Y., Liu R., Yan J., Wang L. (2021). Boost Precision Agriculture with Unmanned Aerial Vehicle Remote Sensing and Edge Intelligence: A Survey. Remote Sens..

[40] Nicolas C., Naila B., Amar R.C. TinyML Smart Sensor for Energy Saving in Internet of Things Precision Agriculture platform. Proceedings of the 2022 Thirteenth International Conference on Ubiquitous and Future Networks (ICUFN).

[41] Viswanatha V., Ramachandra A.C., Hegde P.T., Raghunatha Reddy M.V., Hegde V., Sabhahit V. Implementation of Smart Security System in Agriculture fields Using Embedded Machine Learning. Proceedings of the 2023 International Conference on Applied Intelligence and Sustainable Computing (ICAISC).

[42] Hu N., Su D., Wang S., Nyamsuren P., Qiao Y., Jiang Y., Cai Y. (2022). LettuceTrack: Detection and tracking of lettuce for robotic precision spray in agriculture. Front. Plant Sci..

[43] Dunham M.H. (2002). Data Mining: Introductory and Advanced Topics.

[44] Aggarwal C.C. (2015). Data Mining: The Textbook.

[45] Nodarakis N., Pitoura E., Sioutas S., Tsakalidis A., Tsoumakos D., Tzimas G., Hameurlain A., Küng J., Wagner R., Decker H., Lhotska L., Link S. (2016). kdANN+: A Rapid AkNN Classifier for Big Data. Transactions on Large-Scale Data- and Knowledge-Centered Systems XXIV: Special Issue on Database- and Expert-Systems Applications.

[46] Habib M.T., Raza D.M., Islam M.M., Victor D.B., Arif M.A.I. Applications of Computer Vision and Machine Learning in Agriculture: A State-of-the-Art Glimpse. Proceedings of the 2022 International Conference on Innovative Trends in Information Technology (ICITIIT).

[47] Wu F., Ren Y., Wang X. (2022). Application of Multi-Source Data for Mapping Plantation Based on Random Forest Algorithm in North China. Remote Sens..

[48] Keerthan Kumar T., Shubha C., Sushma S. (2019). Random forest algorithm for soil fertility prediction and grading using machine learning. Int. J. Innov. Technol. Explor. Eng..

[49] Gupta S., Gupta S. (2021). Smart Agriculture and Farming Services Using IoT. Smart Agricultural Services Using Deep Learning, Big Data, and IoT.

[50] Muteba K., Djouani K., Olwal T. Opportunistic Resource Allocation for Narrowband Internet of Things: A Literature Review. Proceedings of the 2020 International Conference on Electrical, Communication, and Computer Engineering (ICECCE).

[51] Tardaguila J., Stoll M., Gutiérrez S., Proffitt T., Diago M.P. (2021). Smart applications and digital technologies in viticulture: A review. Smart Agric. Technol..

[52] Sarri D., Lombardo S., Pagliai A., Perna C., Lisci R., De Pascale V., Rimediotti M., Cencini G., Vieri M. (2020). Smart Farming Introduction in Wine Farms: A Systematic Review and a New Proposal. Sustainability.

[53] Oreški D., Pihir I., Cajzek K. Smart Agriculture and Digital Transformation on Case of Intelligent System for Wine Quality Prediction. Proceedings of the 2021 44th International Convention on Information, Communication and Electronic Technology (MIPRO).

[54] Vela R., Mazarrón F.R., Fuentes-Pila J., Baptista F., Silva L.L., García J.L. (2017). Improved energy efficiency in wineries using data from audits. Ciênc. Téc. Vitiviníc..

[55] Gagliardi G., Lupia M., Cario G., Cicchello Gaccio F., D’Angelo V., Cosma A.I.M., Casavola A. (2021). An Internet of Things Solution for Smart Agriculture. Agronomy.

[56] Chatzisavvas A., Dasygenis M., Louta M. Autonomous Unmanned Ground Vehicle in Precision Agriculture–The VELOS project. Proceedings of the 2022 7th South-East Europe Design Automation, Computer Engineering, Computer Networks and Social Media Conference (SEEDA-CECNSM).

[57] Ponnusamy V., Natarajan S. (2021). Precision agriculture using advanced technology of IoT, unmanned aerial vehicle, augmented reality, and machine learning. Smart Sensors for Industrial Internet of Things: Challenges, Solutions and Applications.

[58] Gialelis J., Theodorou G., Fokaeos M., Karadimas D. An Integrated Low Cost IoT Node based on Discrete Components for Customized Smart Applications; Use case on Precision Agriculture. Proceedings of the 2019 8th Mediterranean Conference on Embedded Computing (MECO).

[59] Ravankar A., Ravankar A.A., Rawankar A., Hoshino Y. (2021). Autonomous and safe navigation of mobile robots in vineyard with smooth collision avoidance. Agriculture.

[60] Martini M., Cerrato S., Salvetti F., Angarano S., Chiaberge M. Position-agnostic autonomous navigation in vineyards with deep reinforcement learning. Proceedings of the 2022 IEEE 18th International Conference on Automation Science and Engineering (CASE).

[61] Liu E., Monica J., Gold K., Cadle-Davidson L., Combs D., Jiang Y. (2023). Vision-based Vineyard Navigation Solution with Automatic Annotation. arXiv.

[62] Valente A., Costa C., Pereira L., Soares B., Lima J., Soares S. (2022). A LoRaWAN IoT System for Smart Agriculture for Vine Water Status Determination. Agriculture.

[63] Pincheira M., Vecchio M., Giaffreda R., Kanhere S.S. (2021). Cost-effective IoT devices as trustworthy data sources for a blockchain-based water management system in precision agriculture. Comput. Electron. Agric..

[64] de Melo D.A., Silva P.C., da Costa A.R., Delmond J.G., Ferreira A.F.A., de Souza J.A., de Oliveira-Júnior J.F., da Silva J.L.B., da Rosa Ferraz Jardim A.M., Giongo P.R. (2023). Development and Automation of a Photovoltaic-Powered Soil Moisture Sensor for Water Management. Hydrology.

[65] Bertocco M., Parrino S., Peruzzi G., Pozzebon A. (2023). Estimating volumetric water content in soil for IoUT contexts by exploiting RSSI-based augmented sensors via machine learning. Sensors.

[66] Meshram A.T., Vanalkar A.V., Kalambe K.B., Badar A.M. (2022). Pesticide spraying robot for precision agriculture: A categorical literature review and future trends. J. Field Robot..

[67] Dai B., He Y., Gu F., Yang L., Han J., Xu W. A vision-based autonomous aerial spray system for precision agriculture. Proceedings of the 2017 IEEE International Conference on Robotics and Biomimetics (ROBIO).

[68] Xue X., Lan Y., Sun Z., Chang C., Hoffmann W.C. (2016). Develop an unmanned aerial vehicle based automatic aerial spraying system. Comput. Electron. Agric..

[69] Becce L., Bloise N., Guglieri G. Optimal Path Planning for Autonomous Spraying UAS framework in Precision Agriculture. Proceedings of the 2021 International Conference on Unmanned Aircraft Systems (ICUAS).

[70] Willocquet L., Clerjeau M. (1998). An analysis of the effects of environmental factors on conidial dispersal of Uncinula necator (grape powdery mildew) in vineyards. Plant Pathol..

[71] Carroll J., Wilcox W. (2003). Effects of humidity on the development of grapevine powdery mildew. Phytopathology.

[72] Kennelly M.M., Gadoury D.M., Wilcox W.F., Magarey P.A., Seem R.C. (2005). Seasonal development of ontogenic resistance to downy mildew in grape berries and rachises. Phytopathology.

[73] Kennelly M.M., Gadoury D.M., Wilcox W.F., Magarey P.A., Seem R.C. (2007). Primary infection, lesion productivity, and survival of sporangia in the grapevine downy mildew pathogen Plasmopara viticola. Phytopathology.

[74] Krasnov H., Cohen Y., Goldshtein E., Ovadia S., Sharon R., Harari A.R., Blank L. (2021). Inconsistent effects of local and landscape factors on two key pests in Israeli vineyards. J. Appl. Entomol..

[75] Szabó M., Csikász-Krizsics A., Dula T., Farkas E., Roznik D., Kozma P., Deák T. (2023). Black Rot of Grapes (Guignardia bidwellii)—A Comprehensive Overview. Horticulturae.

[76] Del Frari G., Oliveira H., Boavida Ferreira R. (2021). White rot fungi (Hymenochaetales) and esca of grapevine: Insights from recent microbiome studies. J. Fungi.

[77] Kenfaoui J., Radouane N., Mennani M., Tahiri A., El Ghadraoui L., Belabess Z., Fontaine F., El Hamss H., Amiri S., Lahlali R. (2022). A panoramic view on grapevine trunk diseases threats: Case of Eutypa dieback, Botryosphaeria dieback, and esca disease. J. Fungi.

[78] Kenfaoui J., Lahlali R., Mennani M., Radouane N., Goura K., El Hamss H., El Ghadraoui L., Fontaine F., Tahiri A., Barka E.A. (2022). Botryosphaeria dieback (Lasiodiplodia viticola): An imminent emerging threat to the moroccan vineyards. Plants.

[79] Mundy D.C., Elmer P., Wood P., Agnew R. (2022). A review of cultural practices for botrytis bunch rot management in New Zealand vineyards. Plants.

[80] Vélez S., Ariza-Sentís M., Valente J. (2023). Mapping the spatial variability of Botrytis bunch rot risk in vineyards using UAV multispectral imagery. Eur. J. Agron..

[81] Bhatia S., Jaffery Z.A., Mehfuz S. A Comparative Study of Wireless Communication Protocols for use in Smart Farming Framework Development. Proceedings of the 2023 3rd International Conference on Intelligent Communication and Computational Techniques (ICCT).

[82] Bhatia S., Jaffery Z.A., Mehfuz S. Development and Analysis of IoT based Smart Agriculture System for Heterogenous Nodes. Proceedings of the 2023 International Conference on Recent Advances in Electrical, Electronics & Digital Healthcare Technologies (REEDCON).

[83] Yu J., Liu W., Song J. C2WSN: A Two-Tier Chord Overlay Serving for Efficient Queries in Large-Scale Wireless Sensor Networks. Proceedings of the 15th International Conference on Advanced Computing and Communications (ADCOM 2007).

[84] Amodu O.A., Othman M. (2018). Machine-to-machine communication: An overview of opportunities. Comput. Networks.

[85] Iraji S., Mogensen P., Ratasuk R. (2017). Recent advances in M2M communications and Internet of Things (IoT). Int. J. Wirel. Inf. Networks.

[86] Pham C., Bounceur A., Clavier L., Noreen U., Ehsan M. (2020). Radio channel access challenges in LoRa low-power wide-area networks. LPWAN Technologies for IoT and M2M Applications.

[87] Ochoa M.N., Guizar A., Maman M., Duda A. Evaluating LoRa energy efficiency for adaptive networks: From star to mesh topologies. Proceedings of the 2017 IEEE 13th International Conference on Wireless and Mobile Computing, Networking and Communications (WiMob).

[88] Adelantado F., Vilajosana X., Tuset-Peiro P., Martinez B., Melia-Segui J., Watteyne T. (2017). Understanding the Limits of LoRaWAN. IEEE Commun. Mag..

[89] Pedregosa F., Varoquaux G., Gramfort A., Michel V., Thirion B., Grisel O., Blondel M., Prettenhofer P., Weiss R., Dubourg V. (2011). Scikit-learn: Machine Learning in Python. J. Mach. Learn. Res..

[90] McKinney W., van der Walt S., Millman J. (2010). Data Structures for Statistical Computing in Python. Proceedings of the 9th Python in Science Conference.

[91] Hunter J.D. (2007). Matplotlib: A 2D graphics environment. Comput. Sci. Eng..

